# On the nature and limits of alkaline earth–triel bonding†

**DOI:** 10.1039/d4sc03832k

**Published:** 2024-09-02

**Authors:** Josef T. Boronski, Liam P. Griffin, Caroline Conder, Agamemnon E. Crumpton, Lewis L. Wales, Simon Aldridge

**Affiliations:** a Chemistry Research Laboratory, Department of Chemistry Oxford OX1 3TA UK josef.boronski@sjc.ox.ac.uk simon.aldridge@chem.ox.ac.uk

## Abstract

The synthesis of a series of isostructural organometallic complexes featuring Ae–Tr bonds (Ae = Be, Mg; Tr = Al, Ga, In) has been investigated, and their electronic structures probed by quantum chemical calculations. This systematic study allows for comparison, not only of the metal–metal bonding chemistries of the two lightest alkaline earth (Ae) elements, beryllium and magnesium, but also of the three triel (Tr) elements, aluminium, gallium, and indium. Computational analyses (NBO, QTAIM, EDA-NOCV) reveal that Be–Tr bonding is more covalent than Mg–Tr bonding. More strikingly, these calculations predict that the beryllium–indyl complex – featuring the first structurally characterised Be–In bond – should act as a source of nucleophilic beryllium. This has been confirmed experimentally by its reactivity towards methyl iodide, which yields the Be–Me functionality. By extension, the electrophilic character of the beryllium centre in the beryllium–gallyl complex contrasts with the umpoled, nucleophilic behaviour of the beryllium centre in both the -indyl and -aluminyl complexes.

## Abstract 2

A series of complexes featuring Ae–Tr bonds (Ae = Be, Mg; Tr = Al, Ga, In) is systematically investigated. The beryllium–indyl complex displays reactivity consistent with a nucleophilic beryllium centre.

## Graphical abstract

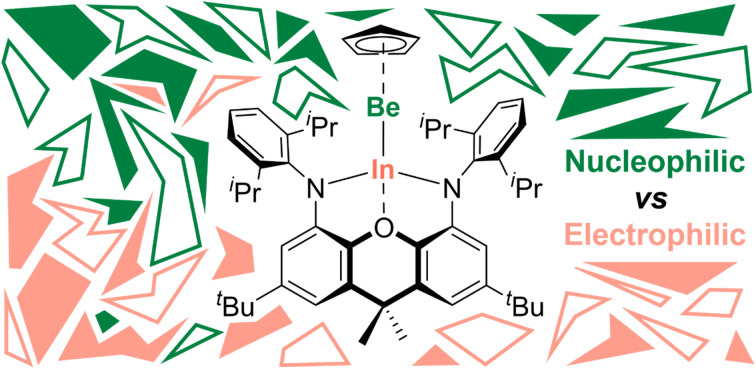

## Introduction

Studies of molecular alkaline earth (Ae) metal chemistry have often overlooked the lightest member of this group, beryllium, primarily due to the high toxicity of the element and its compounds.^1–4^ One particularly neglected facet of molecular beryllium chemistry is beryllium–metal bonding;^5^ until very recently, the only known Be–M bonding combinations were Be–Pt and Be–Al.^6–8^ We have since expanded this to include the homometallic Be–Be bond, as well as Be–Ga and Be–Zn bonding combinations.^9–11^ A stable complex featuring a Be–Mg bond was also reported very recently.^5,12^ Beryllium-containing heterometallic complexes may be useful as single-source precursors for the preparation of new beryllium alloys, which have potential applications in high-end engineering due to their durability and lightness.^13,14^ Additionally, as most fundamental models of chemical bonding are based on the lightest elements, the study of beryllium complexes aids in the validation of these models.^15^ In this context, metal–metal bonding is particularly insightful as the orbital components of such interactions are generally more compositionally nuanced than the ionic interactions of metal cations and “hard” donors.^16,17^

In contrast to the relative paucity of beryllium–metal bonds, studies of the metal–metal bonding of beryllium's heavier homologue, magnesium, are now rather extensive. Indeed, unsupported bonds between magnesium and a range of s-, p-, and d-block metals have been structurally characterised.^18–30^ Such compounds can be conveniently accessed *via* reductive methods, through reaction of metal halides with β-diketiminate-supported magnesium(i) dimers, as has proved successful for the preparation of Mg–Zn, Mg–Cd, and Mg–Mn bonds, amongst others.^20,21,25^ Alternatively, magnesium–metal bonds have also been prepared through reactions of magnesium(ii) reagents with nucleophilic d- or p-block metal complexes.^22,26,31^ More recently, a nucleophilic magnesium(0) complex has been employed for the synthesis of complexes featuring direct Mg–Ae bonds.^18,19^

The chemistry of the group 13 elements (triels; Tr) is dominated by the trivalent state (ns^0^np^0^ valence electronic configuration).^32^ Consequently, triel(iii) compounds typically display electrophilic behaviour due to the large energetic separation between their filled and vacant molecular orbitals. However, in the +1-oxidation state (ns^2^np^0^ valence electronic configuration) – which is generally thermodynamically unstable with respect to disproportionation – the triel elements possess both occupied and unoccupied orbitals in a similar energetic region.^32^ In recent years, a number of nucleophilic aluminyl, gallyl, and indyl anions – formally featuring a triel atom in the +1-oxidation state – have been reported, allowing for the preparation of a diverse range of triel–metal bonds.^33–41^ Owing to the potent σ-electron donor properties of these trielyl metallo–ligands, hetero-bimetallic triel–metal complexes have been found to display novel reactivity patterns. For example, it has been shown that trielyl ligands may induce ‘umpoled’ reactivity (*i.e.*, nucleophilic behaviour) from a variety of electropositive metals (*e.g.*, zinc, copper, silver, and gold).^42–47^

Herein we compare the metal–metal bonding chemistries of beryllium and magnesium by pairing them with triel elements. We report (NON)AlMg(Cp) (AlMg; NON = 4,5-bis(2,6-diisopropylanilido)-2,7-ditert-butyl-9,9-dimethylxanthene) in addition to (NON)InBe(Cp) (InBe) – the first complex with a Be–In bond. Although efforts to prepare (NON)GaMg(Cp) (GaMg) and (NON)InMg(Cp) (InMg) were unsuccessful, these two hypothetical complexes, in addition to AlMg, InBe, and previously reported (NON)AlBe(Cp) (AlBe) and (NON)GaBe(Cp) (GaBe), have been studied by quantum chemical calculations. Computational analyses of this isostructural series suggests that the complete set of Be–Tr bonds is more covalent than all the examined Mg–Tr bonds. Moreover, in line with these calculations, we find that InBe acts as a source of nucleophilic beryllium, similarly to previously reported AlBe. In this context, of the compounds examined here GaBe is exceptional, as it is the only complex with a Tr–Be bond which acts as a beryllium-centred electrophile.

## Results and discussion

We recently reported the syntheses of (NON)AlBe(Cp) (AlBe) and (NON)GaBe(Cp) (GaBe) *via* reactions of BeCp_2_ with {K[E(NON)]}_2_ (E = Al, 1; Ga, 2; NON = 4,5-bis(2,6-diisopropylanilido)-2,7-ditert-butyl-9,9-dimethylxanthene).^10^ Moreover, we demonstrated that AlBe exhibits the reactivity expected of a nucleophilic beryllium complex, while GaBe acts as a gallium-centred nucleophile. Additionally, quantum theory of atoms in molecules (QTAIM) calculations indicate that the Be–Al bond features a non-nuclear attractor (NNA) – that is, a three-dimensional maximum in electron density which is not associated with a nuclear position.^48^ We therefore set out to expand this chemistry to isostructural complexes with Tr–Ae bonds in order to gain greater insight into the nature and limits of such interactions.

**Fig. 1:**
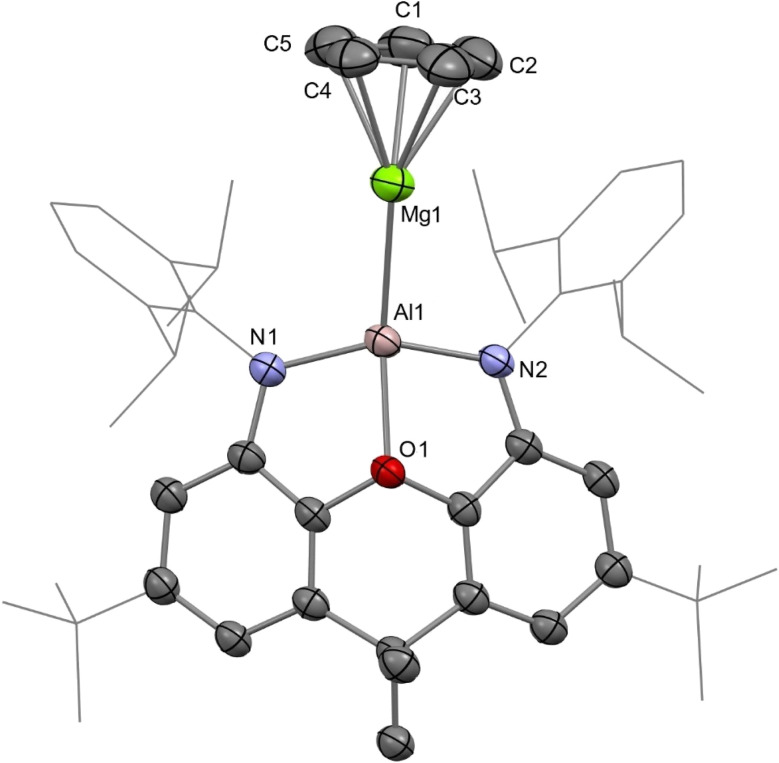
Molecular structure of AlMg in the solid state as determined by X-ray crystallography. Thermal ellipsoids set at 50% probability; hydrogen atoms omitted and selected substituents shown in wireframe format for clarity.

The reaction of 1 with MgCp_2_ quantitatively yields (NON)AlMg(Cp) (AlMg) and KCp, and the structure of the aluminyl complex was confirmed unambiguously by single-crystal X-ray diffraction (SC XRD; Fig. 1). Complex AlMg is the magnesium analogue of previously reported beryllium–aluminyl species AlBe.^10^ Alternatively, AlMg can be described as a half-sandwich magnesium complex, with the magnesium centre bearing cyclopentadienyl and aluminyl ligands. There are ten structurally characterised complexes with Al–Mg bonds, and these feature Al–Mg distances ranging from 2.689(1)–2.7980(6) Å.^6,22,35,46,49–54^ At 2.6575(12) Å, the Al–Mg distance in AlMg is the shortest example of this linkage and is consistent with the sum of the single-bond covalent radii of magnesium and aluminium (2.65 Å).^55^ Notably, Al–Be bonds have also been characterised featuring a wide range of bond lengths (2.310(4)–2.432(6) Å), indicating that the potential energy surfaces for the deformation of both Al–Be and Al–Mg bonds are similarly flat.^6,7,10^ The Al–O distance in AlMg is 2.0185(16) Å and the Al–N distances are 1.890(2) and 1.890(2) Å. For comparison, the Al–O bond length for the potassium aluminyl complex 1 is 2.2792(16) Å and the Al–N bond lengths are 1.963(2) Å and 1.955(2) Å.^35^ The relative shortness of the Al–O and Al–N bond lengths in AlMg suggests the aluminium centre in this complex is less charge rich than that in compound 1. Indeed, upon coordination of the aluminyl ligand to the magnesium centre in the formation of AlMg, a degree of the electron density localised on the aluminium centre in 1 can be considered to be redistributed into the newly formed Al–Mg bond.^10,45^

Complex AlMg was also studied using multinuclear NMR spectroscopy. The ^1^H NMR spectrum of this complex features a cyclopentadienyl ligand resonance at 5.80 ppm, which is somewhat downfield of the analogous signal measured for AlBe (5.15 ppm). Moreover, the carbon atoms of the cyclopentadienyl ligand resonate at 105.7 ppm in the ^13^C{^1^H} NMR spectrum of AlMg, which is also downfield shifted from the corresponding cyclopentadienyl signal measured for AlBe (103.0 ppm).

**Scheme 1:**
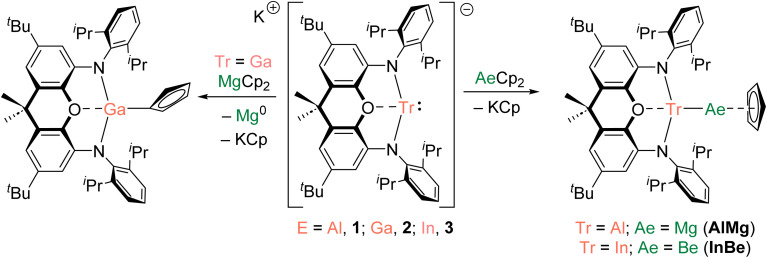
Reactivity of aluminyl (1), gallyl (2), and indyl (3) anions with bis(cyclopentadienyl) magnesium and beryllium.

The preparation of a magnesium analogue of GaBe, (NON)GaMg(Cp) (GaMg), was attempted *via* the reaction of MgCp_2_ and potassium gallyl complex 2.^10^ However, after workup, only the gallium(iii) complex (NON)Ga(η^1^-Cp) was isolated (Scheme 1 and Fig. S11†). A grey precipitate, presumed to contain metallic magnesium, was also formed in this reaction. The apparent reduction of magnesium(ii) by 2 is striking, particularly when this is not observed for the reaction of (more reducing) aluminyl reagent 1 with MgCp_2_.^34^ We hypothesize that the lower bond dissociation energy expected for a Ga–Mg linkage (compared with Al–Mg and Ga–Be bonds) contributes to this divergent reactivity (*vide infra*). Indeed, a small number of complexes with Ga–Mg bonds have previously been prepared, albeit from gallyl anions that are demonstrably less reactive and reducing than 2.^26^

Magnesium–indyl complex (NON)InMg(Cp) (InMg) was targeted through the reaction of MgCp_2_ with (*in situ* generated) indyl anion [(NON)In]K (3), but this yielded intractable product mixtures.^41^ By contrast, the reaction of BeCp_2_ with 3 led to the formation of (NON)InBe(Cp) (InBe) and KCp. Although complex InBe is light sensitive and degrades in solution over the course of 48 hours, it could be isolated in 43% crystalline yield from hexane. This complex represents the first example of a stable molecular species featuring an In–Be bond and expands the range of heterometallic Be–M bonding combinations that have been structurally characterised to six.^6–10,12^ Nevertheless, indium is the third p-block metal to which beryllium has been bonded, after aluminium and gallium, thereby enabling a systematic comparison of the Tr–Be linkage as a function of the triel element. Indeed, the successful preparation of InBe (as well as a previous report of GaBe), and the failed attempts to prepare GaMg and InMg, could be considered qualitative evidence for the more robust nature of Be–M bonds compared with Mg–M bonds.^10^

**Fig. 2:**
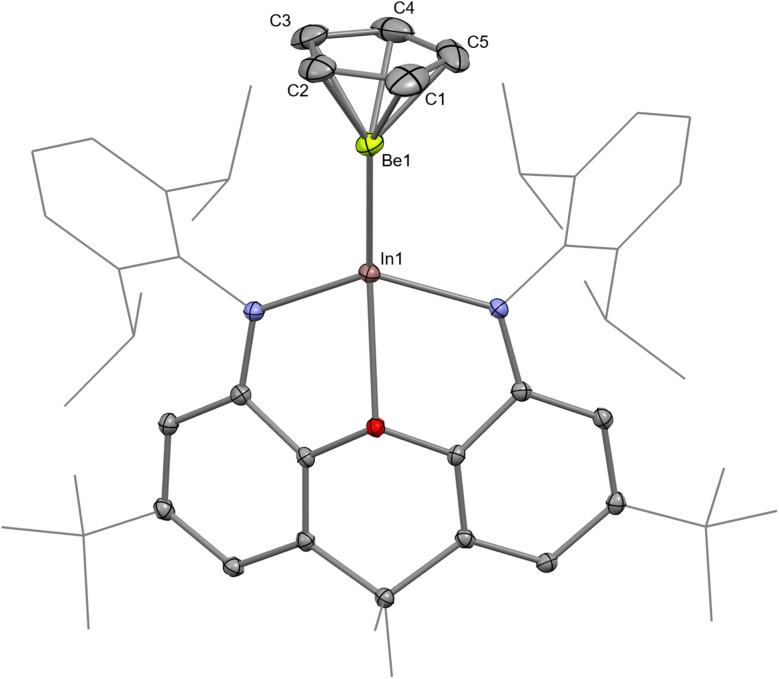
Molecular structure of InBe in the solid state as determined by X-ray crystallography. Thermal ellipsoids set at 50% probability; hydrogen atoms omitted and selected substituents shown in wireframe format for clarity.

The structure of InBe was unambiguously evidenced by SC XRD (Fig. 2). The In–Be distance is 2.358(3) Å, which is significantly shorter than the sum of the single-bond covalent radii of the elements (2.44 Å).^55^ Notably, the In–O and In–N distances in InBe (In–O, 2.4239(14); In–N, 2.1371(17) and 2.1466(17) Å) are significantly shorter than the equivalent metrics reported for potassium indyl complex 3 (In–O, 2.564(2); In–N, 2.342(3), and 2.389(3) Å). This in turn implies that the indium centre in InBe is less charge rich, and thus has a smaller radius, than that in 3. In a similar fashion, the Al–O and Al–N distances in AlBe (Al–O, 2.0813(19); Al–N, 1.893(2) and 1.893(2) Å) are shorter than those measured for potassium aluminyl compound 1 (Al–O, 2.2792(16); Al–N, 1.955(2) and 1.963(2) Å).^10^ The Be-(η^5^-C_5_) centroid distance in InBe is 1.462(2) Å, which is comparable to the analogous metric measured for AlBe (1.498(2) Å). Based on these data, therefore, it appears that the aluminium centre in AlBe and the indium centre in InBe are (partially) oxidised, with concomitant (partial) reduction of the beryllium centre in each complex. Quantum chemical calculations are consistent with this assertion (*vide infra*).

Complex InBe was also probed by multinuclear NMR spectroscopy: its ^1^H NMR spectrum features a cyclopentadienyl ligand resonance at 5.15 ppm, which is essentially identical to the chemical shift measured for AlBe (5.15 ppm). Likewise, the ^13^C{^1^H} NMR spectrum of InBe features a cyclopentadienyl ligand resonance at 102.8 ppm; the equivalent signal for AlBe is measured at 103.0 ppm. The ^9^Be NMR chemical shift measured for a complex of the form CpBeX is a good indicator of the electron density at the beryllium centre: more upfield resonances often correspond to greater electron density at beryllium (Table S2 and Fig. S13†).^56,57^ The ^9^Be NMR chemical shift measured for InBe (−25.6 ppm) is downfield compared with the resonances measured for AlBe (−28.8 ppm) and GaBe (−26.9 ppm), but upfield compared with the signals measured for CpBeCl (−19.5 ppm) and CpBeMe (−20.5 ppm).^57^ This could be taken as evidence that the indyl metallo-ligand, [(NON)In]^−^, is a weaker σ-donor than its aluminyl and gallyl analogues.

**Fig. 3:**
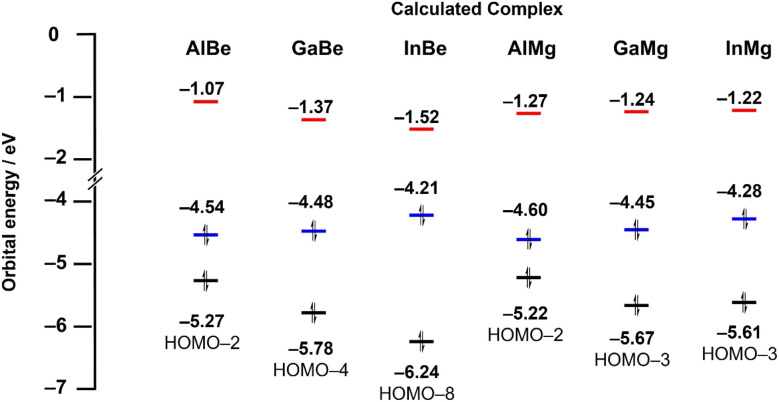
Calculated energies (ωB97X-D4/def2-QZVPP) of key molecular orbitals for AlBe, GaBe, InBe, AlMg, GaMg, and InMg. Red = LUMO energy, blue = HOMO energy, black = energy of principal Tr–Ae bonding orbital (BO).

To gain greater insight into the nature of Tr–Ae bonding, quantum chemical calculations were performed on AlMg and InBe, the hypothetical complexes GaMg and InMg, and the previously reported compounds AlBe and GaBe. All structures were optimised using the r^2^-SCAN-3c composite method, and a single-point calculation was performed on these optimised complexes using the ωB97X-D4 functional with the def2-QZVPP basis set. The optimised geometries of all crystallographically characterised complexes compare well with experimentally determined metrics. For example, the calculated Tr–M bond distances differ from their respective experimentally determined values by less than 0.02 Å, in all cases. The calculated energies of the highest occupied molecular orbital (HOMO), lowest unoccupied molecular orbital (LUMO), and principal Tr–Ae bonding orbital (BO; Fig. S14–S19†) are presented in Fig. 3. In the case of AlBe, GaBe, and InBe, LUMO energy (−1.07, −1.37, and −1.52 eV, respectively) decreases as the atomic number of the triel element is increased, from aluminium, to gallium, to indium. The energy of the BO (−5.27, −5.78, and −6.24 eV, respectively) follows the same trend, with the Tr–Be bonding orbital becoming increasingly stabilised as the triel group is descended. For the same complexes, HOMO energy (−4.54, −4.48, and −4.21 eV, respectively) increases slightly from Al, to Ga, to In. As a result, the energetic separation of the HOMO and LUMO decreases from AlBe, to GaBe, to InBe, and the HOMO-BO separation increases in the same order. By contrast, the LUMO energies of AlMg, GaMg, and InMg (−1.27, −1.24, and −1.22 eV, respectively) exhibit little variance, although HOMO energies (−4.60, −4.45, and −4.28 eV, respectively) for these complexes do increase as the triel group is descended. For the same complexes, however, the energy of the BO does not follow a clear trend; the energy of the principal Ga–Mg and In–Mg bonding orbital is similar (−5.67 and −5.61 eV, respectively), and both are lower in energy than the Al–Mg BO (−5.22 eV).

Natural bond orbital (NBO) calculations were employed to examine the composition of the Tr–Ae interactions in these complexes (Table 1), leading to the discernment of some general trends. The Tr–Be Wiberg bond indices (0.73–0.87) are greater than those calculated for the all of the Tr–Mg bonds (0.62–0.69). In all cases, beryllium makes a greater electron density contribution to Tr–Be bonding (30–39%) than magnesium makes to Tr–Mg bonds (24–28%). Thus, the electron density contributions from the Tr and Ae elements to the Tr–Be bonds are more similar than in Tr–Mg bonds, which might be interpreted as an indicator of greater covalency for the Tr–Be bonds compared to Tr–Mg bonds. Applying the same logic, as a function of the group 13 elements, Ga–Ae bonds are calculated to be the least covalent of all the examined Tr–Ae bonding combinations; the greatest dissimilarity between Tr and Ae contributions to the Tr–Ae bond are calculated for GaBe (70 and 30%, respectively) and GaMg (76 and 24%, respectively). This is consistent with the Pauling electronegativity of gallium (1.81), which is greater than that of aluminium (1.61) and indium (1.78). As an additional point of comparison, the related lithium–aluminyl complex (NON)AlLi(OEt_2_)_2_ (AlLi) was also investigated by NBO methods.^45^ A low Wiberg bond index of 0.33 is calculated for the Al–Li bond within AlLi. Moreover, NBO does not return any data relating to the orbital-based components of Al–Li bonding in AlLi, indicating this interaction is much more ionic than the Al–Mg and Al–Be bonding in AlMg and AlBe, respectively.

**Table 1:** Natural bond orbital calculations for isostructural complexes featuring Tr–Ae bonds^a^

Complex	Wiberg bond index	Contribution to Tr–Ae bond (Tr : Ae)	Charge at Tr (NPA)	Charge at Ae (NPA)	Natural electron configuration at Ae
AlBe	0.82	63 : 37	+1.14	+1.10	[Core] 2s(0.74) 2p(0.16)
GaBe	0.73	70 : 30	+0.90	+1.20	[Core] 2s(0.64) 2p(0.16)
InBe	0.87	61 : 39	+1.28	+0.97	[Core] 2s(0.84) 2p(0.18)
AlMg	0.69	72 : 28	+0.94	+1.35	[Core] 3s(0.60) 3p(0.04)
GaMg	0.62	76 : 24	+0.78	+1.40	[Core] 3s(0.56) 3p(0.04)
InMg	0.69	72 : 28	+0.89	+1.32	[Core] 3s(0.63) 3p(0.04)

a Structures were optimised using the r2-SCAN-3c composite method, and a single-point calculation was performed on these optimised complexes using the ωB97X-D4 functional with the def2-QZVPP basis set.

Quantum theory of atoms in molecules (QTAIM) calculations were also employed to examine the topological profiles of the Tr–Ae bonding within the family of complexes (Tables S3 and S4†). Two metrics are particularly useful when assessing bonding using AIM methods: the electron density at the bond critical point (*ρ*_bcp_; BCP) and the Laplacian of the electron density at the BCP (∇^2^*ρ*_bcp_). A positive ∇^2^*ρ*_bcp_ value corresponds to a local depletion of electron density – a hallmark of non-covalent bonding – while a negative value signifies a local concentration of charge, an indicator of covalent bonding.^58^ In the context of non-covalent bonding (∇^2^*ρ*_bcp_ > 0), total electronic energy density at the BCP (*E*_b_) should also be considered as an additional means of bond classification: previous studies have suggested that if *E*_b_ ≈ 0, bonding is “metallic”; and if *E*_b_ > 0, the interaction is “ionic”.^59,60^

Consistent with our previous analysis, the Al–Be interaction of AlBe is found to feature a non-nuclear attractor (NNA; 3, −3 critical point).^10^ This corresponds to a local three-dimensional maximum in electron density – essentially a “ghost atom” – to which both the beryllium and aluminium centres are “bonded”.^48^ Thus, this complex has Al–NNA and Be–NNA (3, −1) BCPs. None of the other complexes examined here were found to feature a NNA for their respective Tr–Ae interactions. This is possibly because, of the complexes examined here, the Al–Be combination features the two elements with the most spatially concentrated valence orbitals, which overlap particularly efficiently with one another, forming a region of high electron density (*i.e.*, the NNA).^48^ The Al–Be bond of AlBe and In–Be bond of InBe exhibit modest values of *ρ*_bcp_ (Be–NNA, 0.062; Al–NNA, 0.063; In–Be, 0.055 e^−^ bohr^−3^) and negative values of ∇^2^*ρ*_bcp_ (Be–NNA, −0.053; Al–NNA, −0.069; In–Be, −0.021 e^−^ bohr^−5^). This is consistent with the covalent character of these metal–metal bonds.^58,60^ The Ga–Be bond of GaBe might be considered less covalent than the other Tr–Be bonds, as the ∇^2^*ρ*_bcp_ value for the Ga–Be interaction is slightly positive (0.0092 e^−^ bohr^−5^).^59,60^

The Tr–Mg bonds of AlMg, GaMg, and InMg appear to be less covalent than all the Tr–Be bonds examined here; *ρ*_bcp_ values for the Tr–Mg bonds (0.027–0.036 e^−^ bohr^−3^) are all lower than those calculated for the Tr–Be bonds (0.055–0.063 e^−^ bohr^−3^). Moreover, the ∇^2^*ρ*_bcp_ values for all Tr–Mg bonds (0.047–0.078 e^−^ bohr^−5^) are positive, which is a characteristic feature of non-covalent bonding.^58,60^ For all Tr–Mg bonds, *E*_b_ is close to zero (between −0.0026 and −0.0059 a.u.), suggesting these interactions could be described as “metallic” in nature.^59,60^ Additionally, although lithium and beryllium are adjacent in the periodic table, the Al–Li bond of AlLi is far more ionic (*ρ*_bcp_, 0.018 e^−^ bohr^−3^; ∇^2^*ρ*_bcp_, 0.046 e^−^ bohr^−5^) than the Al–Be bonding in AlBe (*ρ*_bcp_: Be–NNA, 0.062; Al–NNA, 0.063 e^−^ bohr^−3^. ∇^2^*ρ*_bcp_: Be–NNA, −0.053; Al–NNA, −0.069 e^−^ bohr^−5^). Indeed, the value of *E*_b_ for the Al–Li bond of AlLi is positive and close to zero (0.00046 a.u.), evidencing an “ionic”/“metallic” description of this interaction.^59,60^

EDA coupled with natural orbitals for chemical valence (EDA-NOCV) calculations were also performed, with all complexes fragmented into two biradicals through homolytic breaking of the metal–metal linkage (Table S6†). A comparison of the magnitudes of eigenvalues for α_1_- and β_1_-pair densities provides an indication as to the polarity of a chemical bond. This analysis reveals the Be–Al bond of AlBe to be the least polarised, with the smallest difference in eigenvalues calculated for the α_1_- and β_1_-pairs of this complex ([CpBe] → [Al(NON)] electron movement, 0.32; [CpBe] ← [Al(NON)] electron movement, 0.38). The Mg–Al bond of AlMg is somewhat more polarized ([CpMg] → [Al(NON)] electron movement, 0.32; [CpMg] ← [Al(NON)] electron movement, 0.47), exhibiting a similar degree of polarisation to the Tr–Be bonds of GaBe and InBe. The Li–Al bond of AlLi is the most polarised of all the linkages examined here, with a large discrepancy between the calculated eigenvalues for the α_1_- and β_1_-pairs ([(Et_2_O)_2_Li] → [Al(NON)] electron movement, 0.20; [CpMg]  ←  [Al(NON)] electron movement, 0.59).

Using EDA-NOCV, we further examined the character of the metal–metal bonds through the comparison of the donor–acceptor fragmentation and homolytic cleavage of the metal–metal bonds (Tables S6 and S7†). This analysis allows for the classification of metal–metal bonding on a spectrum, from dative to polarised covalent to homo-polar/covalent. The most representative bonding model for a given molecule is generally determined by the fragments that yield the lowest magnitude of the orbital interaction energy (*E*_orb_) upon recombination.^11^ The |*E*_orb_| for the homolytic biradical fragmentations of the metal–metal linkages of AlBe, GaBe, AlMg (−79.95, −88.07, and −74.94 kcal mol^−1^, respectively) are smaller than the corresponding |*E*_orb_| for pure donor–acceptor fragmentations of these linkages (Tr→Ae and Ae→Tr), suggesting these Ae–Tr bonds should all be described as “covalent”. However, in the case of both GaBe and AlMg, these linkages are clearly highly polarised as the |*E*_orb_| for the Tr→Ae donor–acceptor fragmentations (−90.18 and −75.38 kcal mol^−1^, respectively) are similar to those of the homolytic fragmentations. For InBe, GaMg, InMg, and AlLi the |*E*_orb_| is smaller for donor–acceptor fragmentation than homolytic cleavage of metal–metal bond, indicating that these complexes may be described as featuring donor–acceptor (Tr→M) bonding.

Natural population analysis (NPA) was performed to evaluate the charge distribution within the selected complexes. In all cases, the charge difference between the Tr and Ae centres within each complex is smaller for those with Tr–Be bonds (0.04–0.31 e^−^) than those with Tr–Mg bonds (0.41–0.62 e^−^). This could also be interpreted as evidence that Tr–Be bonds are more covalent than Tr–Mg bonds. Notably, in the case of AlBe and InBe, beryllium bares a lower positive charge (+1.10 and +0.97 e^−^, respectively), than the Tr element to which it is bonded (+1.14 and +1.28 e^−^, respectively). Voronoi deformation density (VDD) and atomic dipole moment-corrected Hirshfeld (ADCH) charges are also consistent with this Tr(δ+)–Ae(δ–) bond polarity (Table S9†), which suggests that AlBe and InBe could act as sources of nucleophilic beryllium. Additionally, for both complexes, natural electron configuration analysis implies that the valence 2s- and 2p-orbitals of beryllium are populated to an appreciable degree (AlBe, 0.90 e^−^; InBe, 1.02 e^−^). Hence, it could be suggested that an assignment of the +1-oxidation state is appropriate for the beryllium centre in these complexes, as we have previously hypothesized for AlBe, as well as a complex with a Zn–Be bond.^9,10^ If this formalism is applied, the aluminium centre in AlBe and indium centre in InBe would both be assigned the +2-oxidation state. Based on NPA analysis, in the case of all other complexes, the Tr and Ae centres can be assigned the +1- and +2-oxidation states, respectively. The charge distribution calculated for AlLi (Al, +0.76; Li, +0.73 e^−^) and limited population of the lithium valence 2s- and 2p-orbitals (0.26 e^−^) supports an aluminium(i)/lithium(i) formulation for this complex. Hence, the differences between Al–Be and Al–Li bonding appear to be stark, which might be anticipated when considering the far lower Pauling electronegativity of lithium (0.98) compared with beryllium (1.57) and aluminium (1.61). Notwithstanding, although oxidation states may be assigned on the basis of Pauling electronegativities or quantum chemical calculations, these formalisms are only truly useful if they are consistent with the reactivity of a given complex.^10^ Hence, experimental verification of a calculated charge distribution is critical, not least to benchmark computational work.

**Scheme 2:**
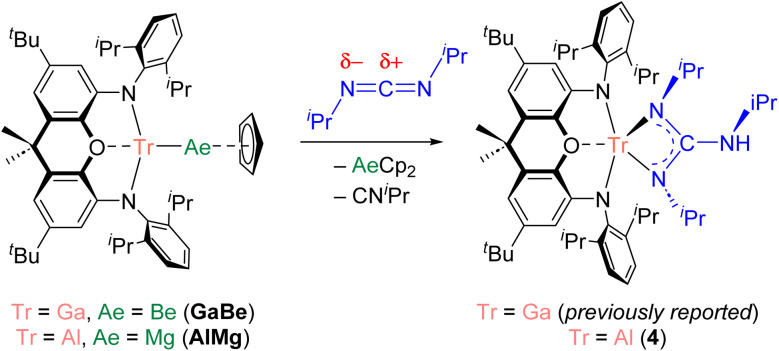
Reactivity of GaBe and AlMg with *N*,*N*-diisopropylcarbodiimide.

We have previously validated the calculated polarity of a variety of aluminium–metal bonds experimentally through their reactions with heteroallenes (*e.g.*, carbodiimides). Carbodiimides feature two nucleophilic nitrogen centres and an electrophilic carbon centre. Thus, the product(s) of the reaction of a carbodiimide with a metal–metal bond provide evidence for the electrophilic and nucleophilic metal centre of the M–M′ linkage, respectively. We previously found that the reaction of AlBe with *N*,*N*′-diisopropylcarbodiimide (CDI) leads to the insertion of the heteroallene into the Al–Be bond, with the formation of Al–N and Be–C bonds.^10^ The connectivity within the product is consistent with the behaviour of a nucleophilic beryllium centre.^44^ By contrast, GaBe was observed to react as a gallium-centred nucleophile with CDI; the initial step of the reaction involves formation of Ga–C and Be–N bonds through insertion of the CDI into the Ga–Be bond.^10^ We therefore set out to obtain experimental evidence for the nature of the bond polarization in novel complexes AlMg and InBe, for which we anticipated the aluminium and beryllium centres, respectively, would behave as the nucleophile. Reaction of AlMg with CDI led to the formation of (NON)Al{(N^i^Pr)_2_C(NH^i^Pr)} (4; Fig. S12†), which is the aluminium analogue of the product of the reaction of GaBe with CDI ([NON]Ga[{N^i^Pr}_2_C{NH^i^Pr}]; Scheme 2).^10^ The analogous reactivity of GaBe and AlMg implies that aluminium acts as the nucleophilic site in the latter, with magnesium behaving electrophilically. Moreover, the data displayed in Table 1 suggest that the polarity of the Al–Mg bond in AlMg (Al, +0.94; Mg, +1.35 e^−^) is very similar to the polarity of the Ga–Be bond in GaBe (Ga, +0.90; Be, +1.20 e^−^). Hence, the equivalent reactivity displayed by these complexes with CDI is not unexpected.

The reaction of InBe with CDI led to the formation of several products, none of which could be conclusively identified. Thus, the reaction of InBe with MeI was examined as an alternative probe reaction; NMR spectroscopy evidenced the formation of CpBeMe (with no signs of CpBeI formation), consistent with the nucleophilic character of the beryllium centre within the starting complex.^35^ Notably, the calculated singlet-triplet gap for InBe is large (41 kcal mol^−1^), indicating that radical reactivity can effectively be ruled out. In contrast to the observed behaviour of InBe, the reaction of AlMg with MeI was found to form (NON)AlMe and CpMgI, which is also consistent with the calculated charge distribution in the starting magnesium–aluminyl complex.^35^

## Conclusions

In summary, new complexes with alkaline earth–triel bonds (NON)AlMg(Cp) (AlMg) and (NON)InBe(Cp) (InBe) have been synthesised. Attempts to prepare (NON)GaMg(Cp) (GaMg) and (NON)InMg(Cp) (InMg) were unsuccessful, despite the fact that the beryllium analogues of these complexes could be isolated. Quantum chemical calculations suggest that the beryllium–triel bonds are more covalent than all the magnesium–triel bonds examined here. Indeed, the Wiberg bond indices for all Be–Tr bonds are greater than those for Mg–Tr bonds, and NBO calculations indicate beryllium makes greater orbital contributions to Ae–Tr interactions, compared with magnesium. In all cases, QTAIM calculations suggest that electron densities at the Ae–Tr bond critical points are greater for Be–Tr bonds than Mg–Tr bonds, which is also an indicator of the higher covalency of Be–Tr interactions. Most notably, in all complexes apart from AlBe and InBe, the positive charge at the alkaline earth metal centre is calculated to be higher than that at the triel centre. Consistently, reactivity studies of AlBe and InBe show that these two systems alone act as sources of nucleophilic beryllium. It is, therefore, striking that the reactivity and calculated charge distribution of GaBe implies that the beryllium centre in this complex is electrophilic. The switch in Tr–Be bond polarity is somewhat unexpected as the Pauling electronegativities of gallium (1.81) and indium (1.78) are similar. Our results highlight the differences between the bonding of both beryllium and magnesium, and provide new insights into the capacity of beryllium for covalent metal–metal bonding.

## Data availability

All data associated with this manuscript are available in the ESI.†

## Author contributions

J. T. B.: conceptualization, investigation (synthetic and computational), visualization, analysis, writing – original draft, writing – review and editing, funding acquisition, supervision, and project administration. L. P. G: investigation (synthetic). C. C: investigation (synthetic) and writing – review and editing. A. E. C.: investigation (computational and crystallographic) and writing – review and editing. L. L. W: investigation (crystallographic). S. A.: funding acquisition, supervision, and writing – review and editing.

## Conflicts of interest

The authors declare no competing interests.

## Supplementary Material

SC-OLF-D4SC03832K-s001

SC-OLF-D4SC03832K-s002
