# Supplementary material for: On the nature and limits of alkaline earth–triel bonding
Source: Chem Sci. 2024 Sep 2;15(37):15377–84. doi: 10.1039/d4sc03832k (PMC11367318; doi:10.1039/d4sc03832k)
Supplement: SC-015-D4SC03832K-s001 [file SC-015-D4SC03832K-s001.pdf]

Supplementary Information (SI) for Chemical Science.
This journal is © The Royal Society of Chemistry 2024

# On The Nature and Limits of Alkaline Earth–Triel Bonding

Josef T. Boronski[a]*, Liam P. Griffin[a], Caroline Conder[a], Agamemnon E. Crumpton[a], Lewis L. Wales[a], Simon Aldridge[a]*

*a*) Chemistry Research Laboratory, Department of Chemistry, Oxford, OX1 3TA, U.K.

## Contents

**General Considerations** – S2

**Synthetic Methods** – S2

**Spectroscopic Data** – S5

**Crystallographic Data** – S10

**$^{9}$Be NMR Chemical Shift Data** – S12

**Computational Details** – S13

Relevant molecular orbitals – S13
QTAIM Calculations – S15
EDA-NOCV Calculations – S20
Misc. Charge Distribution Data – S21
Coordinates of Optimised Structures – S21

**References** – S26

# Experimental Details

## General Considerations

***Health warning:*** *beryllium and its compounds are extremely toxic and can cause irreversible health effects through inhalation or skin contact. The work with beryllium-containing materials described herein was carried out by trained operators, with strict adherence to local and national rules/regulations.*[1]

All manipulations were carried out using Schlenk line or glovebox techniques under an atmosphere of argon or dinitrogen. Solvents were dried by passage through activated alumina towers, dried with $NaK_2$ and degassed before use. Solvents were stored over $NaK_2$. NMR spectra were measured in $C_6D_6$ which was dried over $NaK_2$, with the solvent being distilled under reduced pressure, degassed by three freeze-pump-thaw-cycles and stored under argon in a Teflon valve ampoule. NMR samples were prepared under argon in 5 mm Wilmad 507-PP tubes fitted with J. Young Teflon valves. NMR spectra were measured on a Bruker Avance III HD Nanobay 400 MHz NMR spectrometer equipped with a 9.4 T magnet, Bruker Avance III 500 MHz NMR spectrometer equipped with a 11.75 T magnet, or a Bruker Avance III NMR 500 MHz NMR spectrometer equipped with a 11.75 T magnet and a $^{13}C$ detect cryoprobe. $^1H$ and $^{13}C$ NMR spectra were referenced internally to residual protio-solvent ($^1H$) or solvent ($^{13}C$) resonances and are reported relative to tetramethylsilane (δ = 0 ppm). $^9Be$ NMR spectra were referenced to a 0.43 M solution of $BeSO_4{\cdot}4H_2O$ in $D_2O$ (δ = 0 ppm). Chemical shifts are quoted in δ (ppm) and coupling constants in Hz. Elemental analyses were carried out by London Metropolitan University. The compounds $[K\{Al(NON)\}]_2$ (**1**),[2] $[K\{Ga(NON)\}]_2$ (**2**),[2] $K_2(NON)$, NaCp,[3] and $BeCp_2$ [4] were prepared as described previously. *N,N'*-diisopropylcarbodiimide (CDI) was dried over 3 Å molecular sieves, degassed, distilled, and stored over 3 Å molecular sieves. InCp was purchased and sublimed *in vacuo* before use. $MgCp_2$ (magnesocene) was prepared via a novel synthetic method (see below).

## Synthetic Methods

### *Synthesis of $MgCp_2$*

$MgI_2$ (1.6 g, 5.7 mmol) and NaCp (1.0 g, 11.4 mmol) were combined in a Schlenk flask equipped with a Teflon coated stirrer bar. Toluene (150 mL) was added to the Schlenk flask and the reaction mixture was stirred at 100 °C for a week. The resulting suspension was filtered and the volatiles were removed *in vacuo*. The filtrate was purified by sublimation at 100 °C under reduced pressure to give $MgCp_2$ as a white solid (600 mg, 68%). All data match that reported previously for this compound. $^1H$ NMR (400 MHz, $C_6D_6$, 298 K): δ = 6.01 (s, 10H, $C_5H_5$).[5]

***Synthesis of (NON)Al(MgCp) (AlMg)***

Compound **1** (900 mg, 0.608 mmol) and $MgCp_2$ (188 mg, 1.22 mmol) were combined in a Schlenk flask equipped with a Teflon coated stirrer bar. Toluene (100 mL) was added to the flask and the reaction mixture was stirred overnight at room temperature, leading to the formation of an off-white precipitate. The reaction mixture was filtered and volatiles from the filtrate were removed in vacuo. The resulting waxy off-white solid was dried in vacuo for a further 2 h to remove unreacted $MgCp_2$. The solid was extracted into hot hexane (20 mL) and volatiles were removed *in vacuo* to give (NON)AlMgCp as an off-white powder (0.644 g, 67%). Single crystals suitable for X-ray diffraction experiments were obtained by slow concentration of a hexane solution in a sealed glass λ-type tube. Anal. Calcd for $C_{52}H_{67}AlMgN_2O$: C, 79.32; H, 8.58; N, 3.56. Found: C, 79.14; H, 8.31; N, 3.29. $^1$H NMR (400 MHz, $C_6D_6$, 298 K): δ = 1.14 (d, $^3J_{HH}$ = 6.8 Hz, 12 H, CH(C$H_3$)$_2$), 1.21 (s, 18 H, C(C$H_3$)$_3$), 1.25 (d, $^3J_{HH}$ = 6.8 Hz, 12 H, CH(C$H_3$)$_2$), 1.59 (s, 6H, XA-C(C$H_3$)$_2$), 3.39 (sept., $^3J_{HH}$ = 6.8 Hz, 4H, C$H$(CH$_3$)$_2$), 5.80 (s, 5H, $C_5H_5$), 6.38 (d, $^4J_{HH}$ = 2.0 Hz, 2H, XA-*o*-C*H*), 6.76 (d, $^4J_{HH}$ = 2.0 Hz, 2H, XA-*p*-C*H*), 7.27 (s, 6H, ArH). $^{13}$C{$^1$H} NMR (101 MHz, $C_6D_6$, 298 K): δ = 23.0, 25.0 (CH(*C*H$_3$)$_2$), 25.7 (XA-*C*(CH$_3$)$_2$), 29.0 (*C*H(CH$_3$)$_2$), 31.8 (C(*C*H$_3$)$_3$), 35.2 (*C*(CH$_3$)$_3$), 36.9 (XA-C(*C*H$_3$)$_2$), 105.7 (*C*$_5$H$_5$), 107.6, 110.2, 124.3, 124.9, 126.7, 126.9, 141.9, 147.1, 148.2, 149.0 (Ar-*C*).

***Synthesis of (NON)In(BeCp) (InBe)***

*Procedure conducted in the absence of light.* A solid mixture of $K_2$(NON) (30 mg, 0.040 mmol) and InCp (7.2 mg, 0.04 mmol) was added to an ampoule fitted with a Teflon valve and equipped with a glass-coated stirrer bar. Benzene (1 mL) was condensed into the vessel *in vacuo* at –196 °C. The mixture was allowed to warm to room temperature and stirred for 10 minutes. Subsequently, $BeCp_2$ (5.6 mg, 0.040 mmol) in benzene (1 mL) was added to the reaction flask. The bright-yellow solution and metallic precipitate was stirred for one hour. Subsequently, volatiles were removed *in vacuo*, and soluble material was dissolved in hexane (0.5 mL). The suspension was filtered and volatiles were removed *in vacuo*, leaving a bright yellow powder. Yield: 15 mg, 43%. Single crystals suitable for X-ray diffraction experiments were obtained by slow concentration of a hexane solution in a sealed glass λ-type tube. Complex **InBe** is sensitive to light and degrades in solution over the course of 48 hours. Anal. Calcd for $C_{52}H_{67}BeInN_2O$: C, 72.63; H, 7.85; N, 3.26. Found: C, 72.86; H, 7.99; N, 3.29. $^1$H NMR (400 MHz, $C_6D_6$, 298 K): δ = 1.09 (d, $^3J_{HH}$= 7.0 Hz, 12H, CH(C$H_3$)$_2$), 1.22 (d, $^3J_{HH}$= 6.9 Hz, 12H, CH(C$H_3$)$_2$), 1.31 (s, 18H, C(C$H_3$)$_3$), 1.67 (s, 6H, C(C$H_3$)$_2$), 3.45 (sept, $^3J_{HH}$= 6.5 Hz, 4H, C$H$(CH$_3$)$_2$), 5.15 (s, 5H, C$_5H_5$), 6.38 (d, $^4J_{HH}$= 2.5 Hz 2H, XA-*o*-C*H*), 6.78 (d, $^4J_{HH}$= 2.5 Hz 2H, XA-*p*-C*H*), 7.23 (m, 6H, Ar*H*); $^9$Be NMR (42 MHz, $C_6D_6$): δ = –25.6 ($w_{1/2}$ = 21.7 Hz) $^{13}$C{$^1$H} NMR (101 MHz, $C_6D_6$): δ = 24.2, 24.4 (CH(*C*H$_3$)$_2$), 24.9 (*C*(CH$_3$)$_2$), 28.4 (*C*H(CH$_3$)$_2$), 28.5 (C(*C*H$_3$)$_3$), 31.3 (*C*(CH$_3$)$_3$), 31.7 (C(*C*H$_3$)$_2$), 102.8 (*C*$_5$H$_5$), 107.7, 109.1, 123.9, 125.8, 129.7, 142.4, 145.7, 146.0, 147.4, 149.8 (Ar-*C*).

***Synthesis of (NON)Ga(η$^1$-Cp)***

A solid mixture of **2** (80 mg, 0.051 mmol) and $MgCp_2$ (16 mg, 0.10 mmol) was added to an ampoule fitted with a Teflon valve and equipped with a Teflon-coated stirrer bar. Benzene (1 mL) was condensed into the vessel *in vacuo* at –196 °C. The mixture was allowed to warm to room temperature and stirred for 10 minutes. Subsequently, volatiles were removed *in vacuo*, and soluble material was dissolved in hexane (0.5 mL). The suspension was filtered and the resulting yellow solution was slowly concentrated to 0.2 mL, forming colourless crystals of (NON)Ga(η$^1$-Cp). Yield: 5.8 mg, 7%. Single crystals suitable for X-ray diffraction experiments were obtained by slow concentration of a hexane solution in a sealed glass λ-type tube. Anal. calcd for $C_{52}H_{67}GaN_2O$: C, 77.51; H, 8.38; N, 3.48. Found: C, 77.42; H, 8.24; N, 3.22. $^1$H NMR (400 MHz, $C_6D_6$, 298 K): 1.14 (d, $^3J_{HH}$ = 7.0 Hz, 12 H, CH(C$H_3$)$_2$), 1.17 (d, $^3J_{HH}$ = 7.0 Hz, 12 H, CH(C$H_3$)$_2$), 1.24 (s, 18 H, C(C$H_3$)$_3$), 1.68 (s, 6H, XA-C(C$H_3$)$_2$), 3.47 (sept., $^3J_{HH}$ = 7.0 Hz, 4H, C$H$(CH$_3$)$_2$), 5.93 (s, 5H, $C_5H_5$), 6.52 (d, $^4J_{HH}$ = 2.2 Hz, 2H, XA-*o*-C*H*), 6.99 (d, $^4J_{HH}$ = 2.2 Hz, 2H, XA-*p*-C*H*), 7.23 (s, 2H, Ar*H*), 7.25 (s, 2H, Ar*H*). As the product was not isolable from the reaction mixture in bulk quantities, satisfactory $^{13}$C{$^1$H} NMR data could not be obtained.

***Synthesis of (NON)Al{(N$^i$Pr)$_2$C(NH$^i$Pr)} (4)***

Compound **AlMg** (40 mg, 0.051 mmol) was added to a Schlenk flask equipped with a Teflon-coated stirrer bar. Benzene (1 mL) and *N,N'*-diisopropylcarbodiimide (0.1 mL) was condensed into the vessel *in vacuo* at –196 °C. The mixture was allowed to warm to room temperature and stirred overnight, forming a brown solution. Subsequently, volatiles were removed *in vacuo*, and soluble material was dissolved in hexane (0.5 mL). The brown solution was filtered and slowly concentrated to 0.2 mL, forming colourless crystals of **4**. Yield: 30 mg, 61%. Anal. calcd. for $C_{63}H_{98}AlN_5O$: C, 78.13; H, 10.20; N, 7.23. Found: C, 78.06; H, 9.84; N, 7.17. $^1$H NMR (400 MHz, $C_6D_6$, 298 K): δ = 0.06 (d, $^3J_{HH}$ = 6.5 Hz, 3H, NCH(C$H_3$)$_2$), 0.81 (d, $^3J_{HH}$ = 6.5 Hz, 3H, NCH(C$H_3$)$_2$), 1.11 (d, $^3J_{HH}$ = 6.5 Hz, 6H, NCH(C$H_3$)$_2$), 1.23 (m, 24H, ArCH(C$H_3$)$_2$), 1.27 (s, 18H, C(C$H_3$)$_3$), 1.31 (d, $^3J_{HH}$ = 6.5 Hz, 6H, NCH(C$H_3$)$_2$), 1.68 (s, 3H, C(C$H_3$)$_2$), 1.87 (s, 3H, C(C$H_3$)$_2$), 2.41 (sept., $^3J_{HH}$ = 6.5 Hz, 1H, NC$H$(CH$_3$)$_2$), 3.60 (sept., $^3J_{HH}$ = 6.8 Hz, 2H, ArC$H$(CH$_3$)$_2$), 3.81 (sept., $^3J_{HH}$ = 6.5 Hz, 1H, NC$H$(CH$_3$)$_2$), 3.95 (sept., $^3J_{HH}$ = 6.8 Hz, 2H, ArC$H$(CH$_3$)$_2$), 4.12 (sept., $^3J_{HH}$ = 6.5 Hz, 1H, NC$H$(CH$_3$)$_2$), 6.17 (d, $^4J_{HH}$ = 2.0 Hz, 2H, XA-*o*-C*H*), 6.76 (d, $^4J_{HH}$ = 2.0 Hz, 2H, XA-*p*-C*H*), 7.26-7.36 (m, 6H, Ar*H*). $^{13}$C{$^1$H} NMR (101 MHz, $C_6D_6$, 298 K): δ = 23.1 (NCH(*C*H$_3$)$_2$), 25.0, 25.1, 25.2, 25.9 (CH(*C*H$_3$)$_2$), 26.2, 26.5 (*C*H(CH$_3$)$_2$), 27.5, 29.0 (NCH(*C*H$_3$)$_2$), 31.9 (C(*C*H$_3$)$_3$), 32.0 (*C*(CH$_3$)$_3$), 35.2 (C(*C*H$_3$)$_2$), 37.0 (*C*(CH$_3$)$_2$), 43.9, 44.1, 45.1 (N*C*H(CH$_3$)$_2$), 106.1, 111.2, 124.3, 124.4, 125.9, 131.7, 139.3, 144.7, 145.1, 147.3, 147.6, 148.4 (Ar-*C*), 160.0 (N*C*N).

## Spectroscopic Data

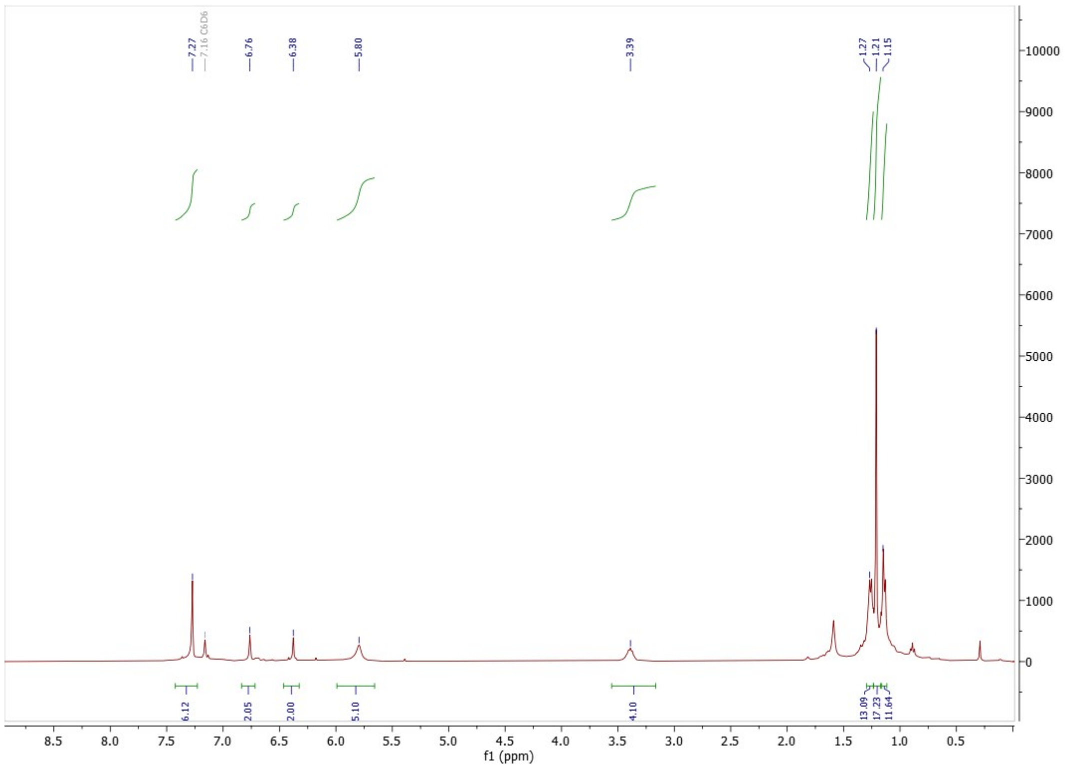


Figure S1: $^{1}H$ NMR spectrum of compound **AlMg** in $C_6D_6$.

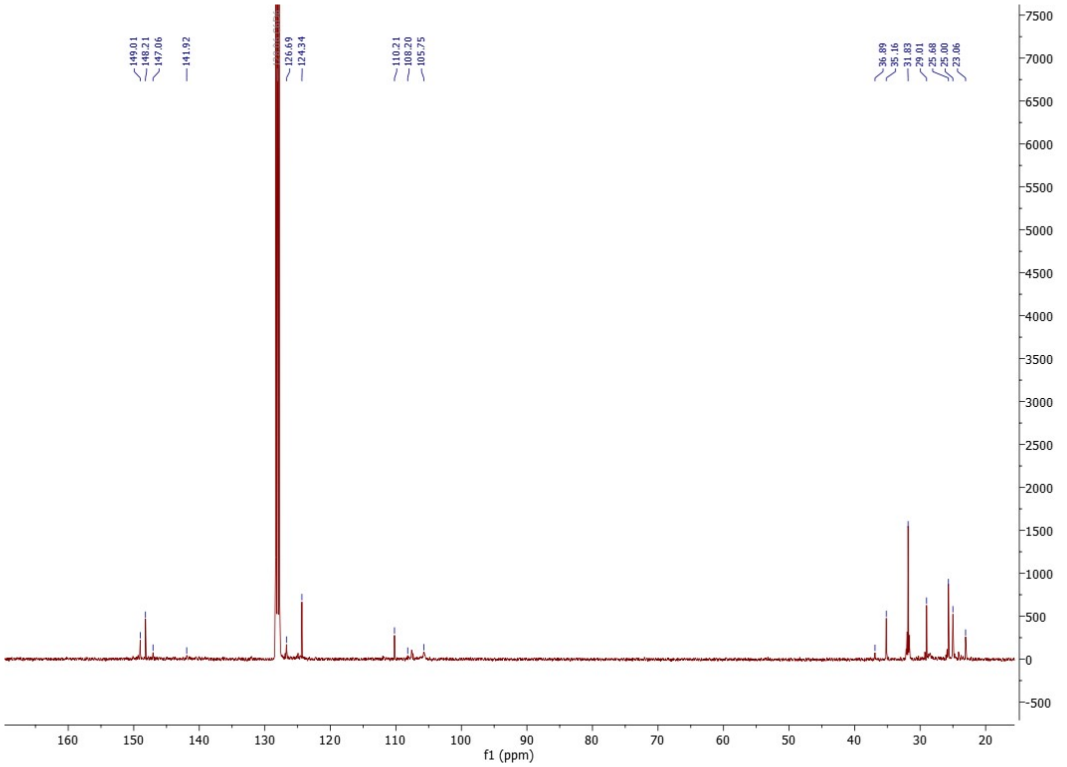


Figure S2: $^{13}C$ {$^{1}H$} NMR spectrum of compound **AlMg** in $C_6D_6$.

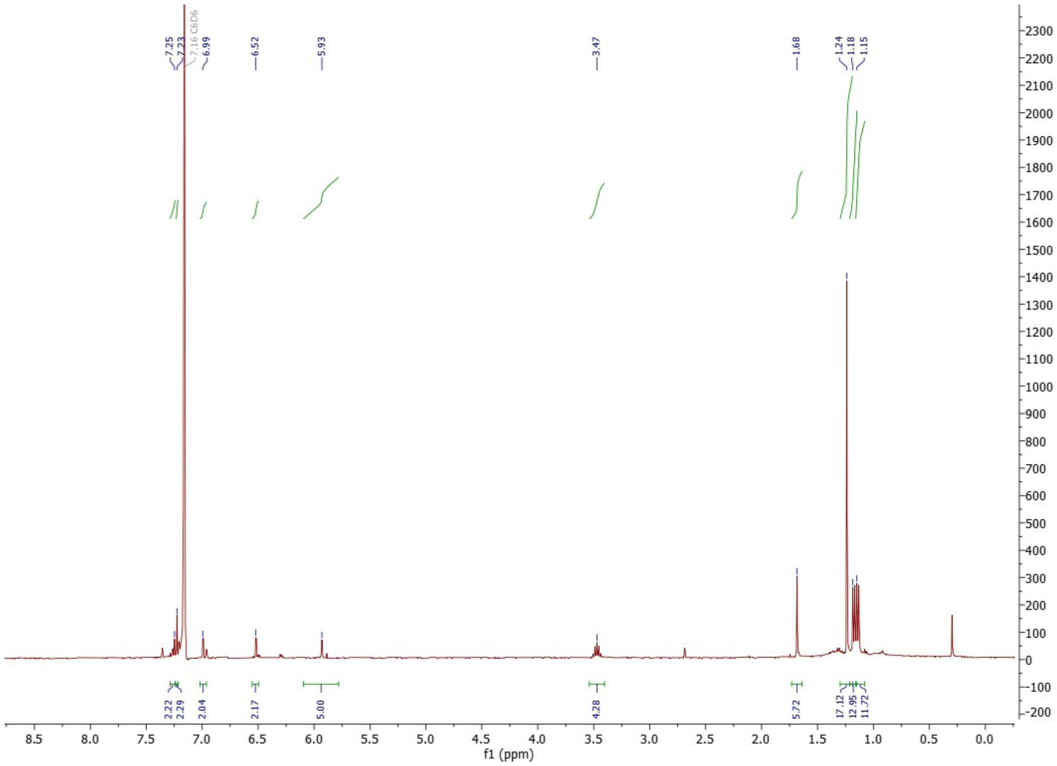


Figure S3: $^{1}$H NMR spectrum of (NON)Ga($\eta^1$-Cp) in $C_6D_6$.

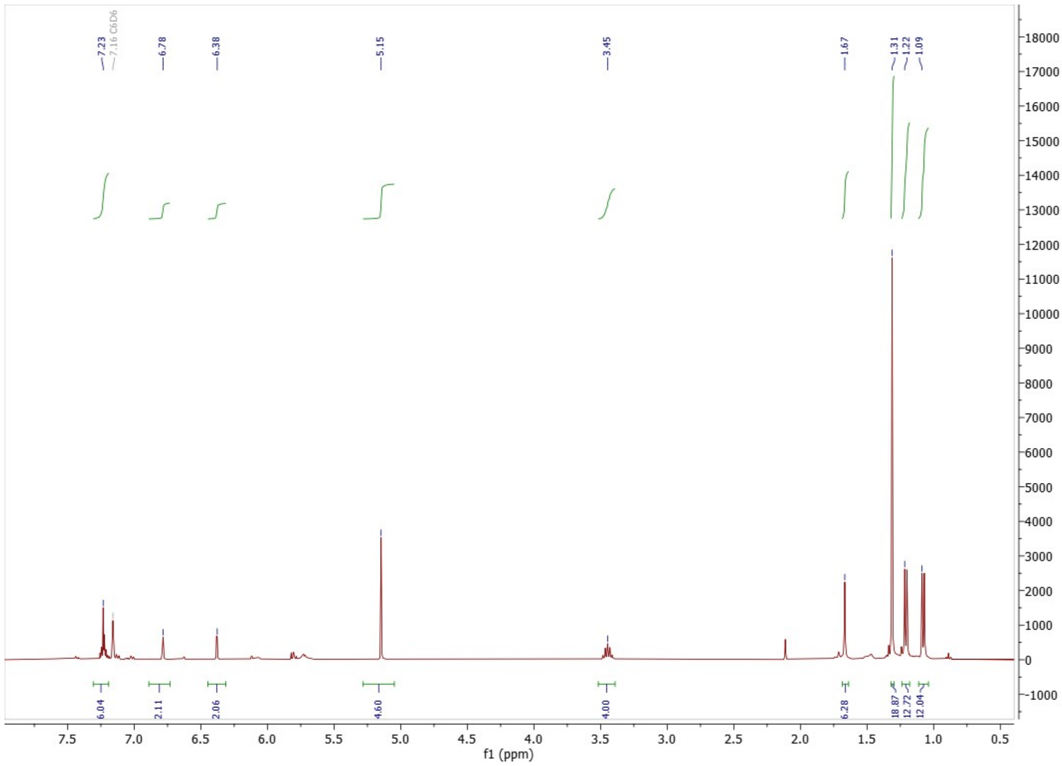


Figure S4: $^{1}$H NMR spectrum of compound **InBe** in $C_6D_6$.

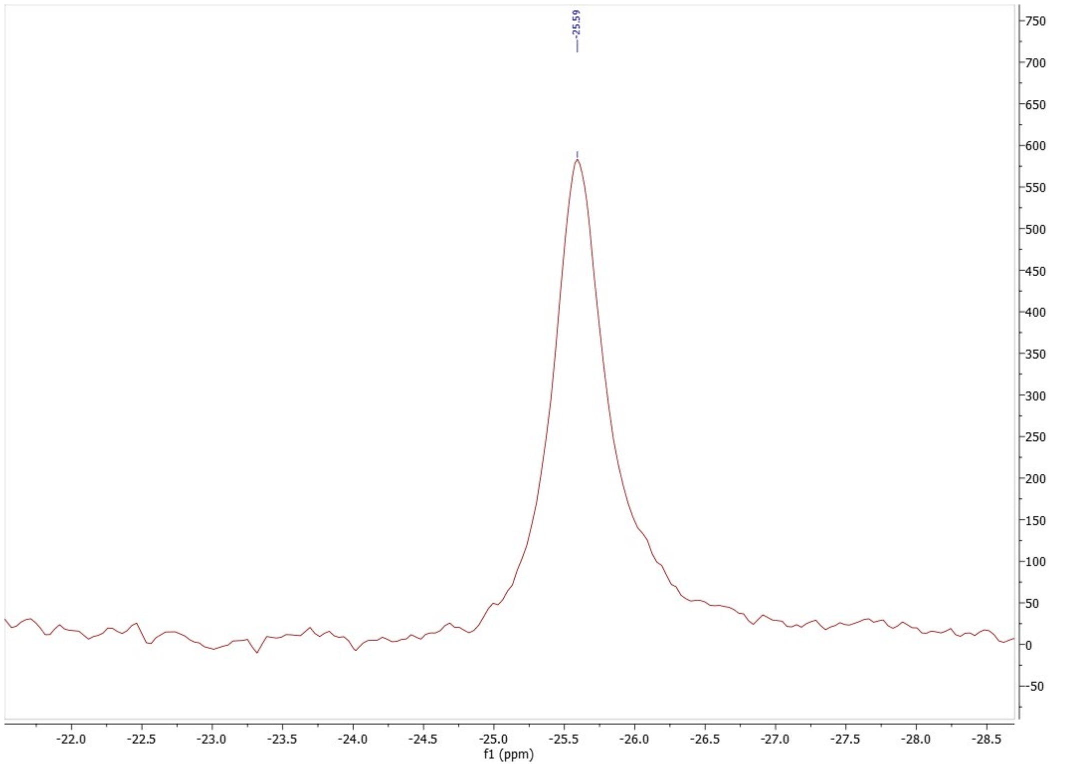


Figure S5: $^{9}$Be NMR spectrum of compound **InBe** in $C_6D_6$.

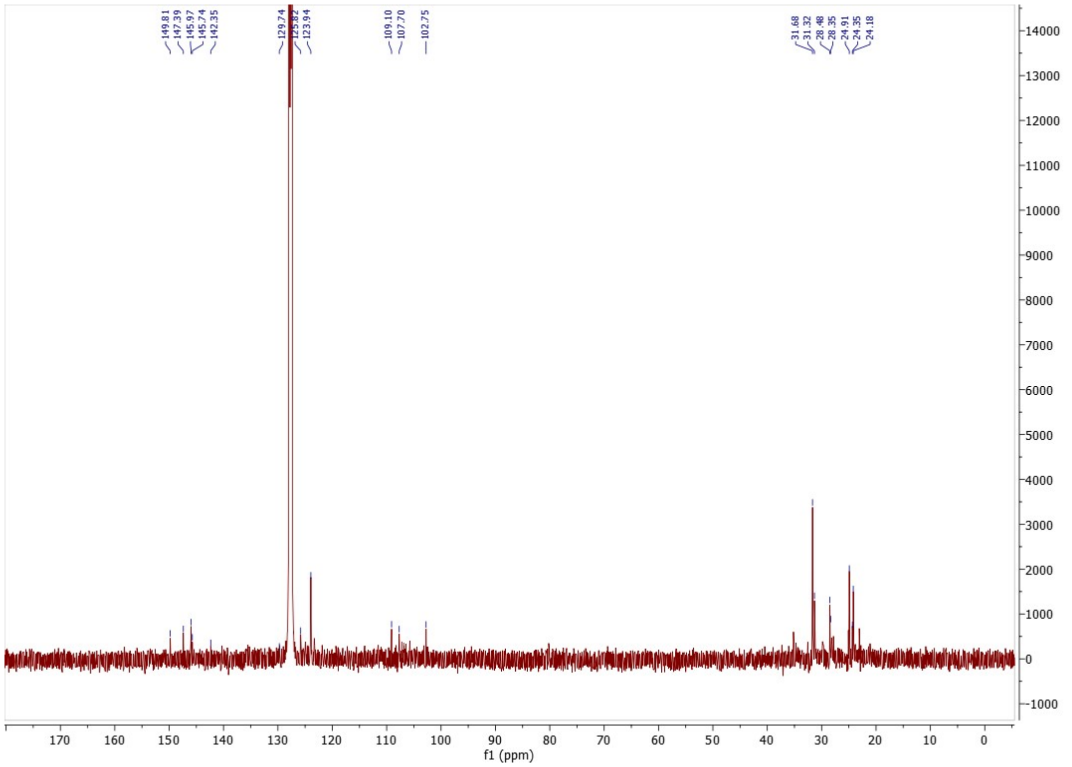


Figure S6: $^{13}$C {$^{1}$H} NMR spectrum of compound **InBe** in $C_6D_6$.

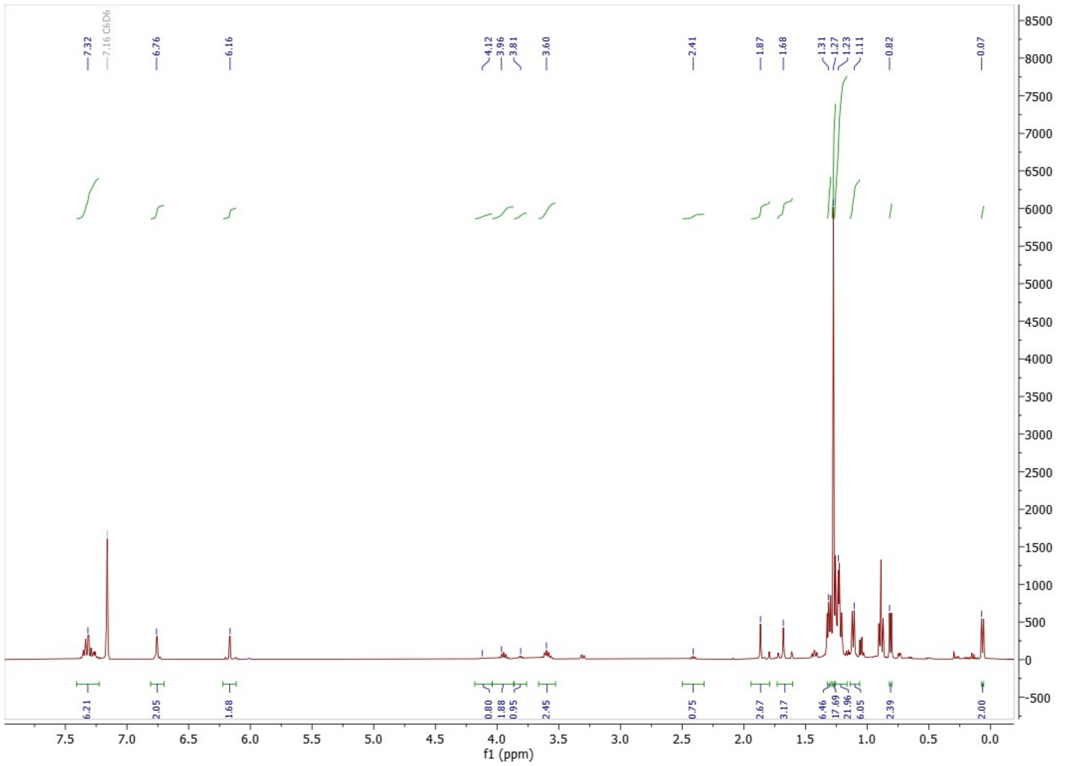


Figure S7: $^{1}H$ NMR spectrum of compound **4** in $C_6D_6$.

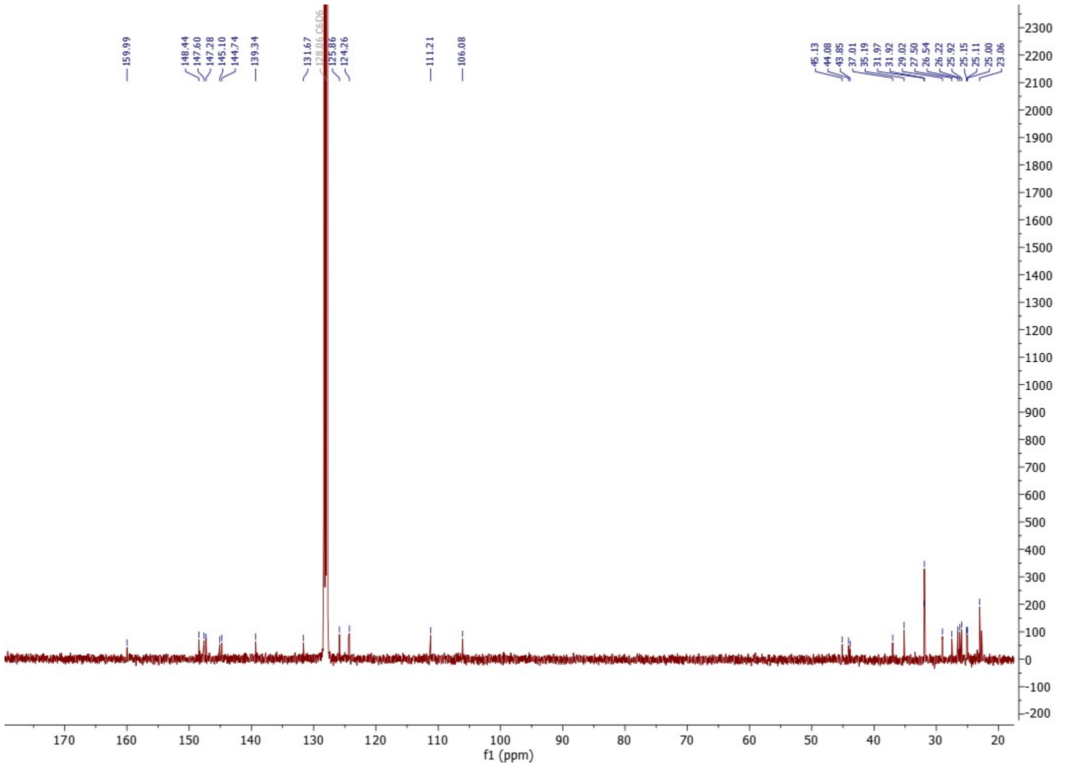


Figure S8: $^{13}C$ {$^{1}H$} NMR spectrum of compound **4** in $C_6D_6$.

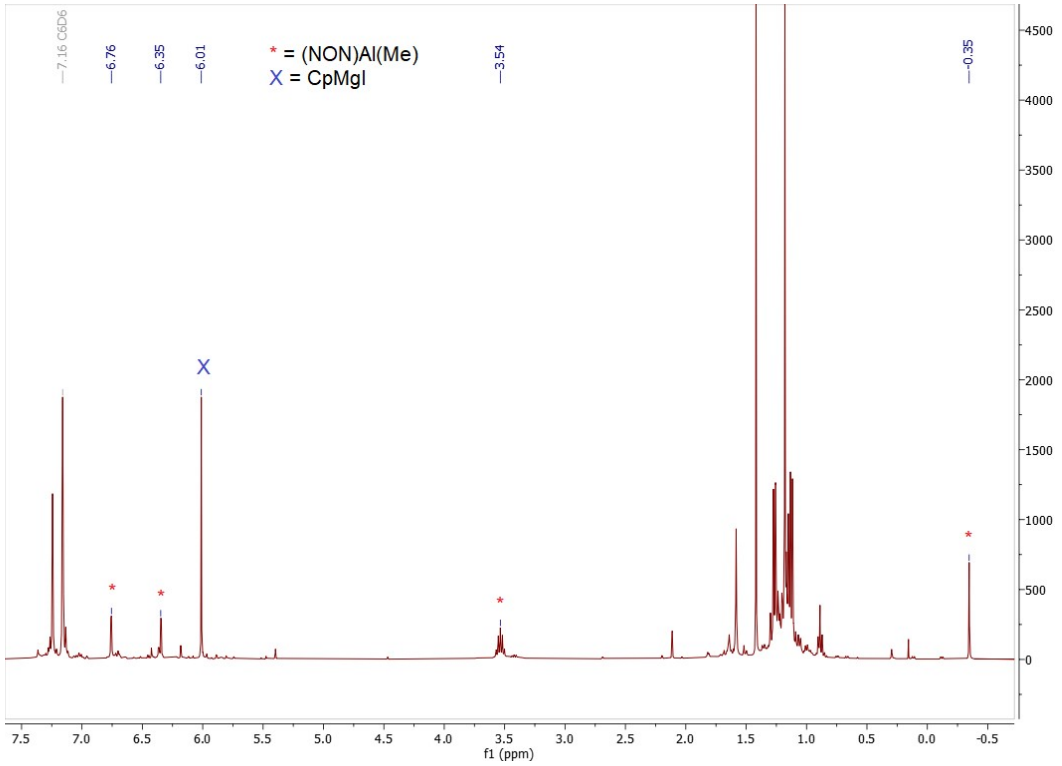


Figure S9: $^{1}$H NMR spectrum of reaction of **AlMg** with MeI in $C_6D_6$.

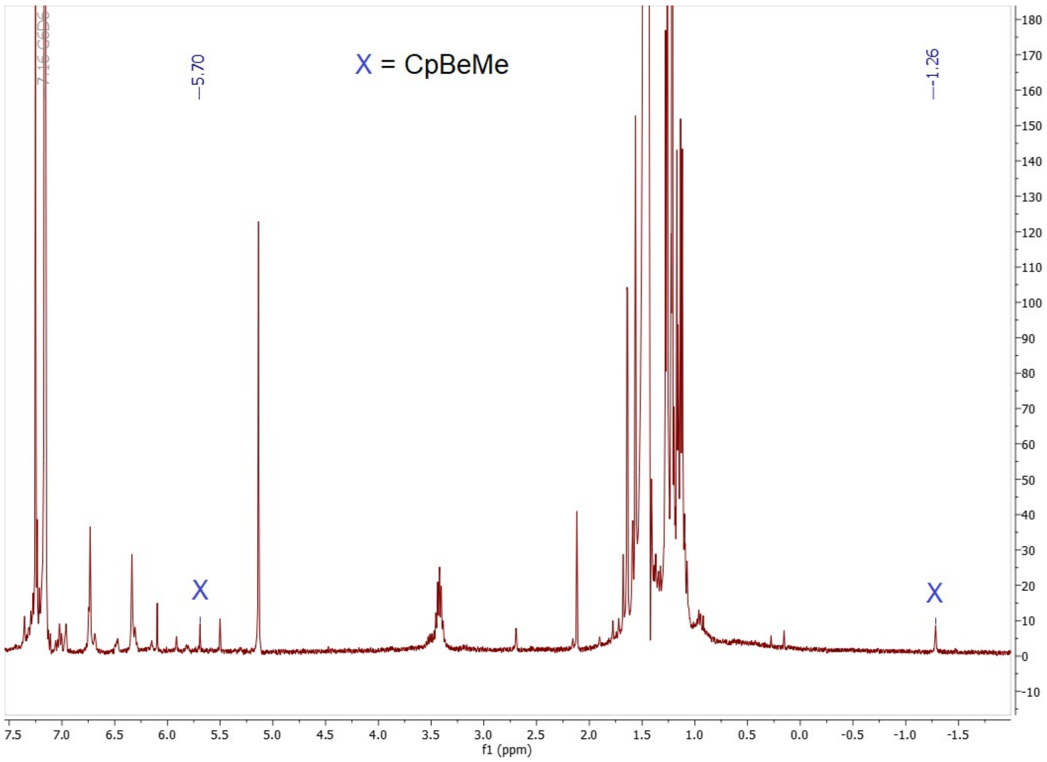


Figure S10: $^{1}$H NMR spectrum of reaction of **InBe** with MeI in $C_6D_6$.

## Crystallographic Data

Crystallographic data for **AlMg**, (NON)Ga($\eta^1$-Cp), **InBe**, and **4** were collected using an Oxford Diffraction/Agilent SuperNova or Rigaku XtaLAB Synergy-R. Crystals were selected under Paratone-N or perfluorinated oil, mounted on MiTeGen Micromount loops and quench-cooled using an Oxford Cryosystems open flow $N_2$ cooling device.[6] Selected details of data collection are given in Table 1. Data collected were processed using the CrysAlisPro package, including unit cell parameter refinement and inter-frame scaling (which was carried out using SCALE3 ABSPACK within CrysAlisPro).[7] Equivalent reflections were merged and diffraction patterns processed with the CrysAlisPro suite. Structures were solved *ab initio* from the integrated intensities using SHELXT and refined on F2 using SHELXL with the graphical interface OLEX2.[8–10] Crystallographic data is given in the supplementary deposited CIF files (CCDC 2346890-2346893) and can be obtained free of charge from the Cambridge Crystallographic Data Centre *via* http://www.ccdc.cam.ac.uk/data_request/cif.

Table S1: Selected X-ray data collection and refinement parameters

| | **AlMg** | **(NON)Ga(Cp)** | **InBe** | **4** |
|---|---|---|---|---|
| Formula | C52 H67 Al Mg N2 O | C52 H67 Ga N2 O | C52 H67 Be In N2 O | C63 H98 Al N5 O |
| Fw (g mol$^{-1}$) | 787.36 | 805.79 | 859.90 | 968.44 |
| Cell setting | monoclinic | monoclinic | monoclinic | monoclinic |
| Space group | $P2_1/c$ | C2/c | $P2_1/c$ | $P2_1/c$ |
| *a* (Å) | 13.6415(2) | 19.3033(2) | 13.9427(2) | 13.87645(13) |
| *b* (Å) | 19.3339(3) | 17.3028(2) | 19.1025(3) | 21.59607(19) |
| *c* (Å) | 18.9730(3) | 27.4999(4) | 18.6052(3) | 20.08526(15) |
| *α* (°) | 90 | 90 | 90 | 90 |
| *β* (°) | 109.1377(17) | 103.2039(12) | 108.451(2) | 102.7343(8) |
| *γ* (°) | 90 | 90 | 90 | 90 |
| *V* (Å$^{-3}$) | 4727.45(13) | 8942.17(19) | 4700.59(14) | 5871.03(9) |
| *Z* | 4 | 8 | 4 | 4 |
| $\rho_{calc}$ (g cm$^{-3}$) | 1.106 | 0.085 | 1.215 | 1.096 |
| Radiation, λ (Å) | 1.54184 | 0.71073 | 1.54184 | 1.54184 |
| μ (mm$^{-1}$) | 0.778 | 0.654 | 4.288 | 0.621 |
| $R_{(int)}$ | 0.0908 | 0.0747 | 0.0401 | 0.0383 |
| Parameters | 530 | 556 | 530 | 813 |
| $R_1$ (all data/$I$ >2σ($I$)) | 0.0661 | 0.0607 | 0.0313 | 0.0585 |
| $\omega R_2$ (all data/$I$ >2σ($I$)) | 0.2016 | 0.1667 | 0.0806 | 0.1800 |
| GooF | 1.044 | 1.163 | 1.038 | 1.017 |
| *T* (K) | 100.0(2) | 100.0(2) | 150.0(2) | 150.0(2) |
| CCDC Deposition No. | 2346890 | 2346893 | 2346892 | 2346891 |

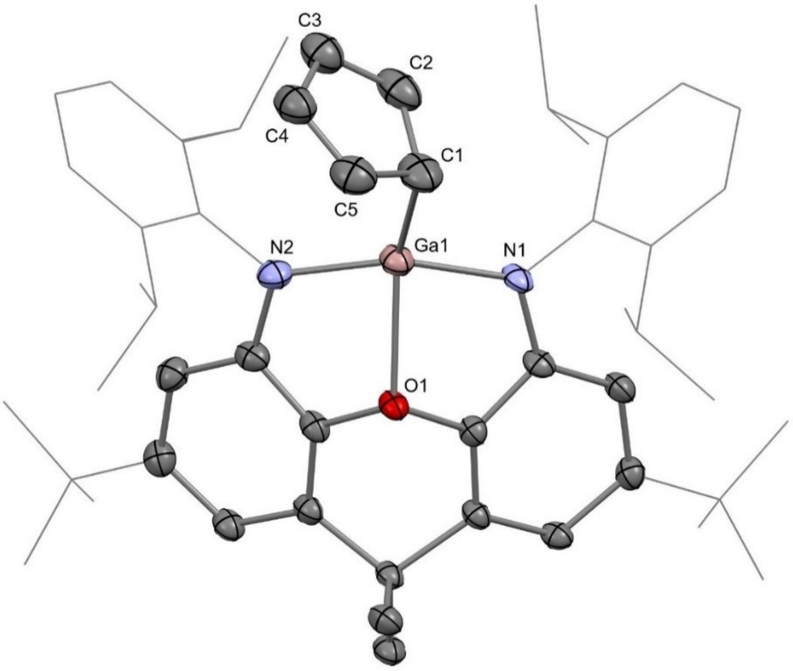


Figure S11: Molecular structure of (NON)Ga($\eta^1$-Cp) in the solid state as determined by X-ray crystallography. Thermal ellipsoids set at 50% probability; hydrogen atoms omitted and selected substituents shown in wireframe format for clarity.

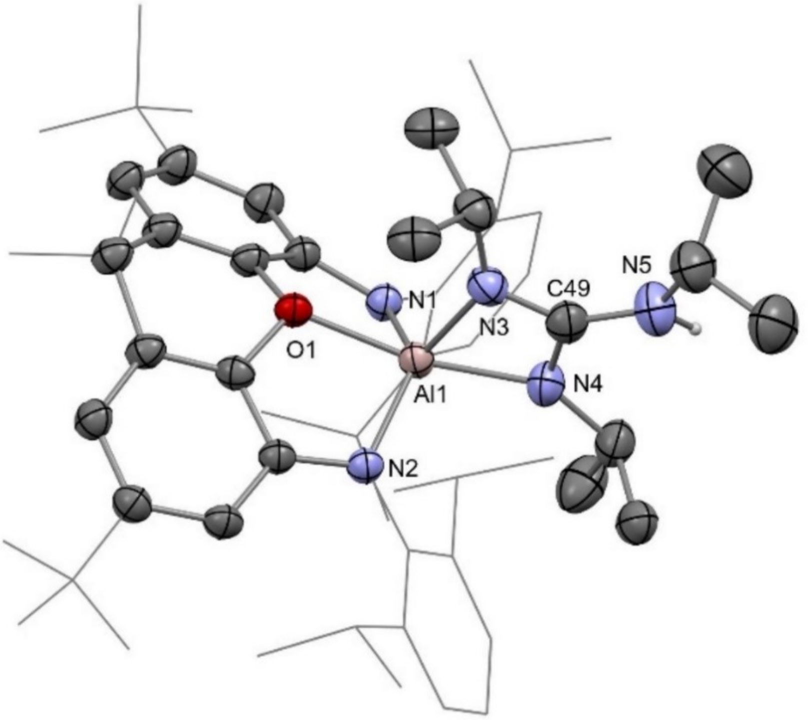


Figure S12: Molecular structure of compound **4** in the solid state as determined by X-ray crystallography. Thermal ellipsoids set at 50% probability; residual solvent and selected hydrogen atoms omitted for clarity. Selected substituents shown in wireframe format for clarity.

## $^{9}$Be NMR Chemical Shifts – CpBeR Complexes

$^{9}$Be NMR chemical shift is very sensitive to the degree of electron density at a particular beryllium centre.[11,12] Of relevance here is the $^{9}$Be NMR chemical shift of a cyclopentadienyl-coordinated beryllium centre. Increasingly upfield $^{9}$Be NMR signals correspond to beryllium centres which are more electron-rich/reduced.[13]

Table S2: $^{9}$Be NMR chemical shifts (in $C_6D_6$) for a range of CpBeR species and the Pauling electronegativity of the atom from each group which is bonded to beryllium.[11]

| R-Group (CpBeR) | $\delta_{9Be}$ / ppm | Pauling Electroneg. of Donor Atom |
|---|---|---|
| [(NON)Al] | –28.8 | 1.61 |
| ($^{Dipp}$Nacnac)Zn | –27.7 | 1.65 |
| $Me_3Si$ | –27.7 | 1.90 |
| CpBe | –27.6 | 1.57 |
| [$Me_2(SiMe_3)Si$] | –27.2 | 1.90 |
| [(NON)Ga] | –26.9 | 1.81 |
| [(NON)In] | –25.6 | 1.78 |
| [{NON}Al{(N$^{i}$Pr)$_2$C}] | –24.6 | 2.55 |
| Me | –20.5 | 2.55 |
| Cl | –19.5 | 3.16 |
| Br | –19.5 | 2.96 |

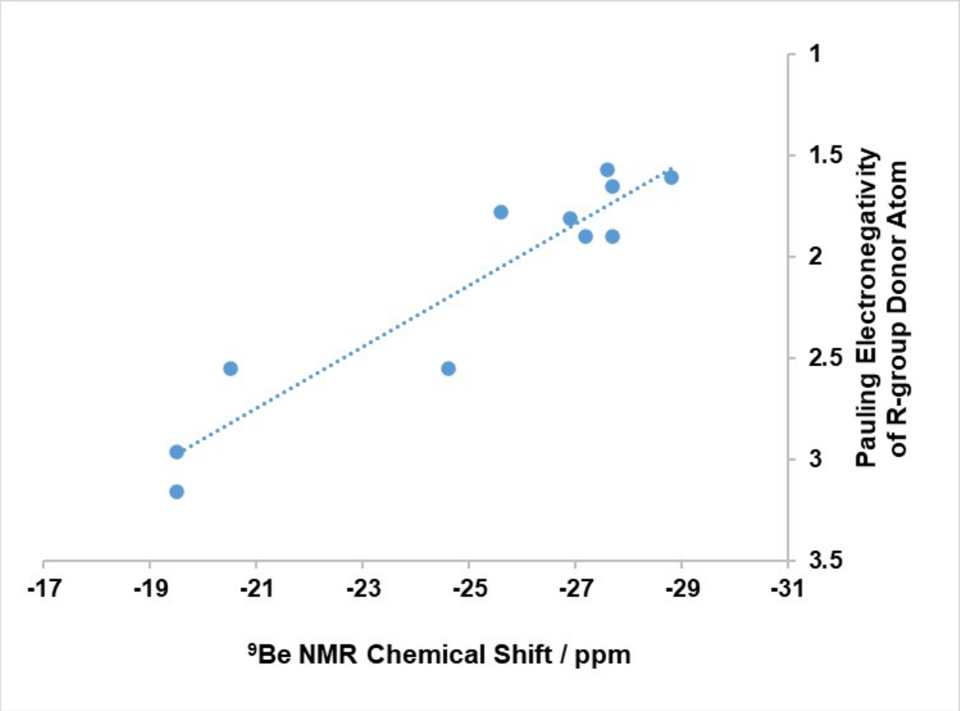


Figure S13: Plot of $^{9}$Be NMR chemical shifts for a range of CpBeR species *vs* the Pauling electronegativity of the atom from each group which is bonded to beryllium.

## Computational Details

The structures of complexes **AlBe**, **GaBe**, **InBe**, **AlMg**, **GaMg**, **InMg**, and **AlLi** were optimised using ORCA (Revision 5.0.4).[14,15] Specifically, R2-SCAN was used,[16,17] in conjunction with the Def2-TZVPPm basis set with the D4 dispersion correction,[18] employing the geometrical counterpoise correction gCP (together known as the R2SCAN-3c method).[19,20] Subsequently, a single point calculation was performed on this optimised structure with the ωB97X range-separated hybrid functional,[21,22] in conjunction with the Def2-QZVPP basis set [23,24] and the D4 dispersion correction.[18] The nature of the stationary points (minima) was confirmed by full frequency calculations, and are characterized by zero imaginary frequencies. Natural bond orbital was performed on the ORCA wavefunction using NBO 7.0.[25] Quantum Theory of Atoms in Molecules calculations were performed using the ORCA wavefunctions for the respective complexes, and were generated using Multiwfn 3.8.[26] EDA-NOCV was performed using Multiwfn 3.8 and ORCA (Revision 5.0.4) at the M062x-def2-TZVP level for comparisons with our previous work.

*Relevant Molecular Orbitals*

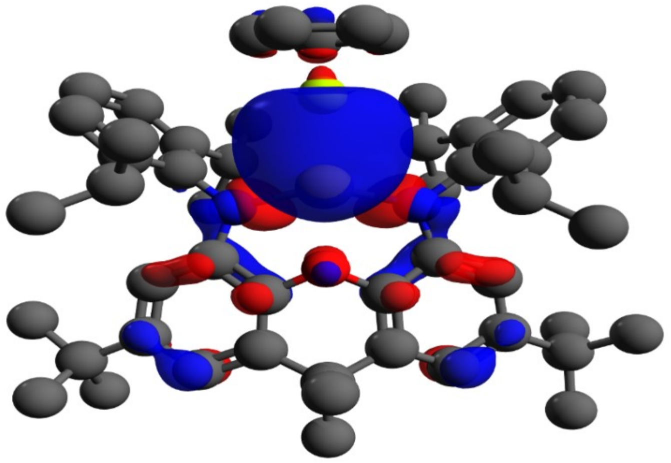

Figure S14: Principal Al–Be bonding orbital (HOMO–2) of **AlBe** (0.03 a.u.).

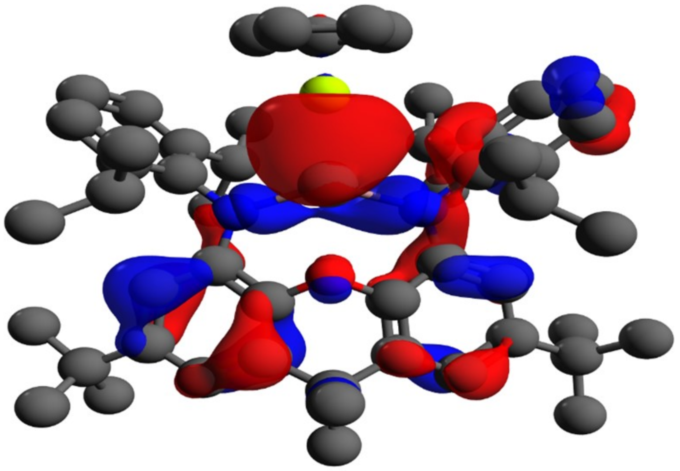

Figure S15: Principal Ga–Be bonding orbital (HOMO–4) of **GaBe** (0.03 a.u.).

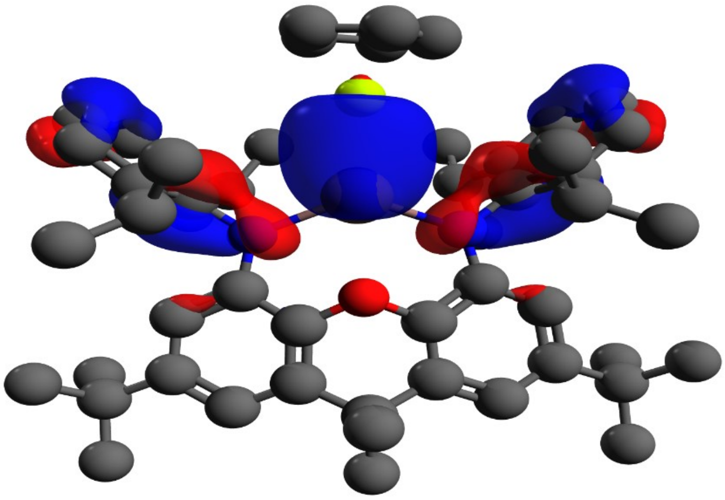

Figure S16: Principal In–Be bonding orbital (HOMO–8) of **InBe** (0.03 a.u.).

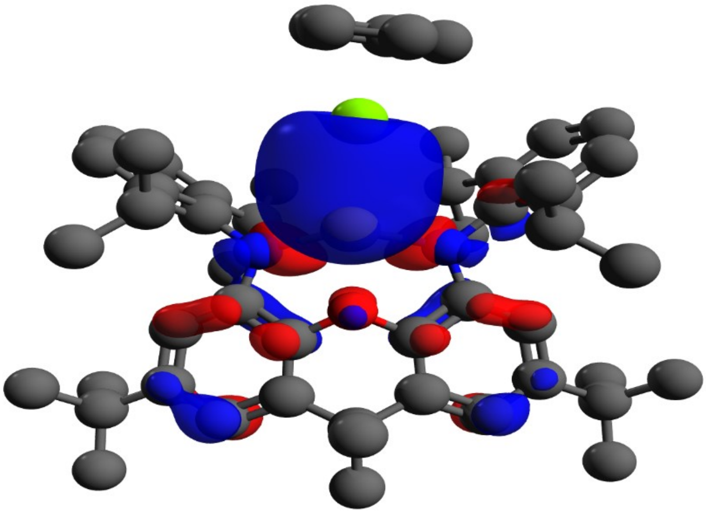

Figure S17: Principal Al–Mg bonding orbital (HOMO–2) of **AlMg** (0.03 a.u.).

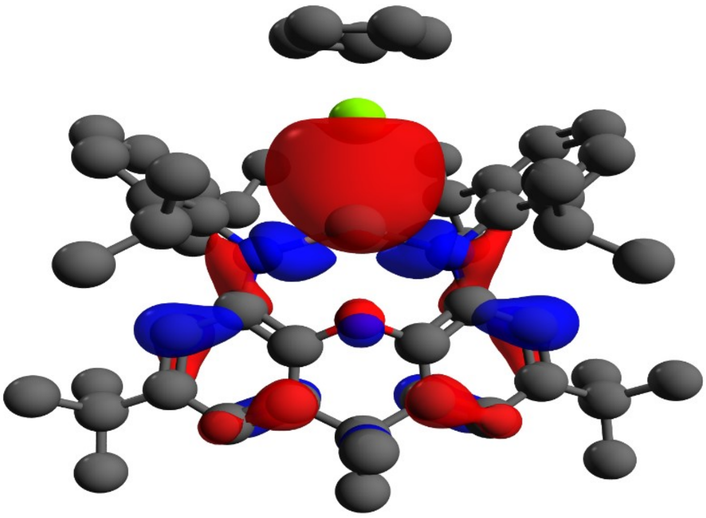

Figure S18: Principal Ga–Mg bonding orbital (HOMO–3) of **GaMg** (0.03 a.u.).

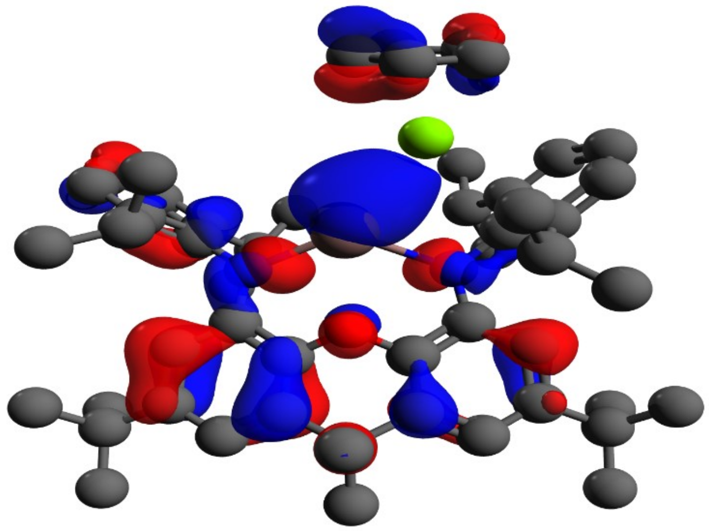

Figure S19: Principal In–Mg bonding orbital (HOMO–3) of **InMg** (0.03 a.u.).

*Quantum Theory of Atoms in Molecules Calculations*

Table S3: Charge distribution (Bader) calculated for complexes examined in this study.

| | Tr | Ae | NNA |
|---|---|---|---|
| **AlBe** | 2.056106 | 1.401902 | -0.960639 |
| **GaBe** | 0.568247 | 1.494281 | - |
| **InBe** | 0.599657 | 1.443632 | - |
| **AlMg** | 1.143892 | 1.434668 | - |
| **GaMg** | 0.59264 | 1.524848 | - |
| **InMg** | 0.586081 | 1.605489 | - |

Table S4: Summary of QTAIM data generated for **AlBe, GaBe, and InBe**. All values in a.u., unless explicitly stated.

| | **AlBe** | | | **GaBe** | **InBe** |
|---|---|---|---|---|---|
| **Critical Point** | **Be-NNA** | **Al-NNA** | **NNA** | **Ga-Be** | **In-Be** |
| **$\rho_{bcp}$ / e$^-$ Bohr$^{-3}$** | 0.062 | 0.063 | 0.063 | 0.061 | 0.055 |
| **Lag. $E_k$** G(r) | 0.023 | 0.014 | 0.0030 | 0.034 | 0.024 |
| **Hamilt. $E_k$** K(r) | 0.036 | 0.031 | 0.027 | 0.032 | 0.030 |
| **Pot. E Density** V | -0.058 | -0.045 | -0.030 | -0.066 | -0.054 |
| **Energy Density** $E_b$ | -0.036 | -0.031 | -0.027 | -0.032 | -0.030 |
| **$\nabla^2\rho_{bcp}$ / e$^-$ Bohr$^{-5}$** | -0.053 | -0.069 | -0.095 | 0.0092 | -0.021 |
| **ELF** | 0.599 | 0.808 | 0.989 | 0.385 | 0.467 |
| **LOL** | 0.550 | 0.672 | 0.906 | 0.442 | 0.484 |

Table S5: Summary of QTAIM data generated for **AlMg**, **GaMg**, **InMg**, and **AlLi**. All values in a.u., unless explicitly stated.

| | **AlMg** | **GaMg** | **InMg** | **AlLi** |
|---|---|---|---|---|
| **Critical Point** | **Al-Mg** | **Ga-Mg** | **In-Mg** | **Al-Li** |
| **$\rho_{bcp}$ / e⁻ Bohr⁻³** | 0.034 | 0.036 | 0.027 | 0.018 |
| **Lag. $E_k$** G(r) | 0.018 | 0.024 | 0.016 | 0.011 |
| **Hamilt. $E_k$** K(r) | 0.0059 | 0.0045 | 0.0026 | -0.00046 |
| **Pot. E Density** V | -0.024 | -0.029 | -0.019 | -0.011 |
| **Energy Density** $E_b$ | -0.0059 | -0.0045 | -0.0026 | 0.00046 |
| **$\nabla^2\rho_{bcp}$ / e⁻ Bohr⁻⁵** | 0.047 | 0.078 | 0.054 | 0.046 |
| **ELF** | 0.257 | 0.180 | 0.157 | 0.0972 |
| **LOL** | 0.371 | 0.319 | 0.301 | 0.247 |

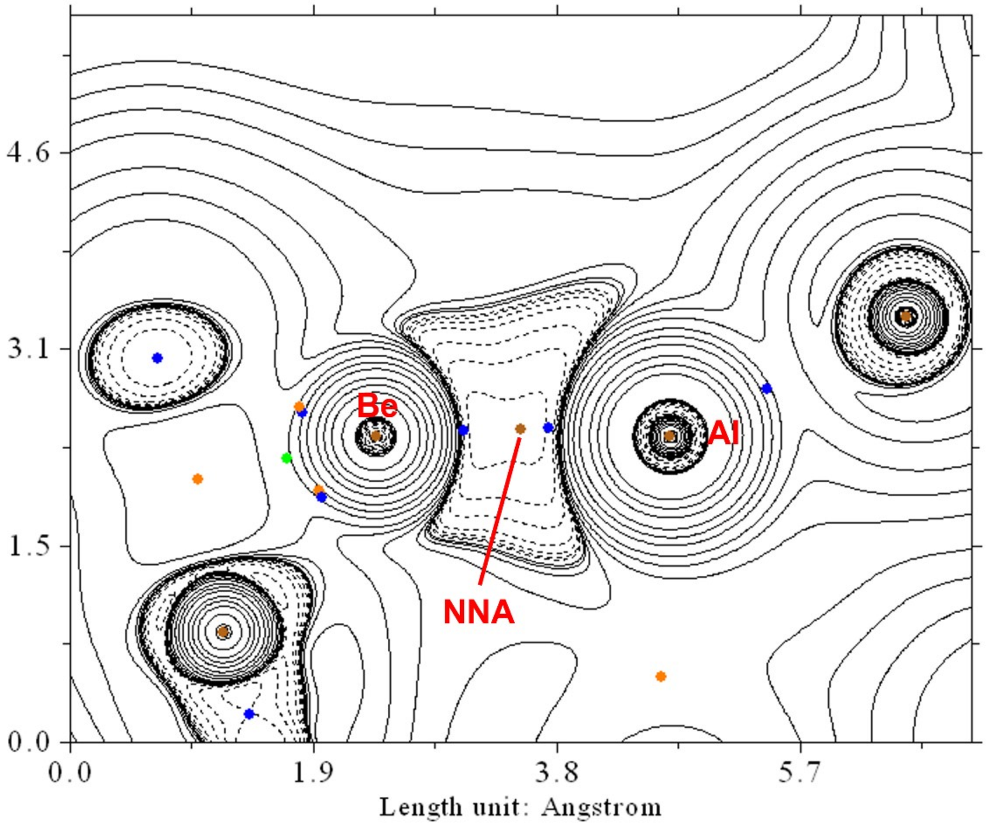


Figure S20: Laplacian of electron density contour plot for **AlBe**. Blue dots are (3, –1) points (BCP), brown dots are (3, –3) points (nucleus/NNA).

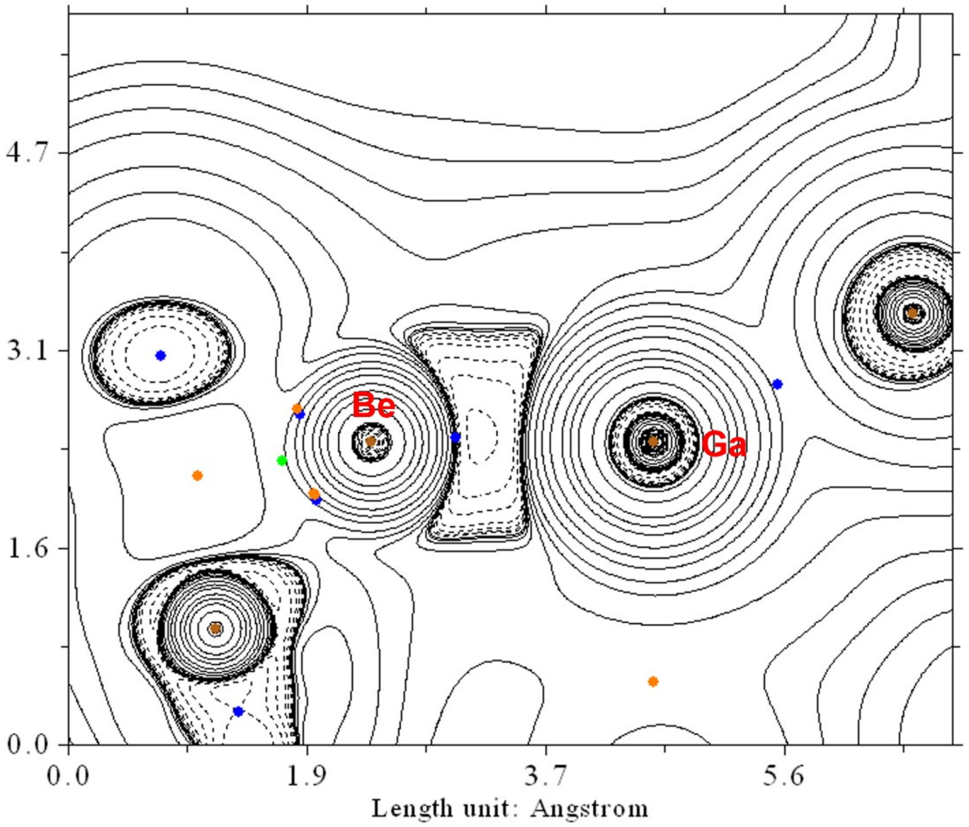


Figure S21: Laplacian of electron density contour plot for **GaBe**. Blue dots are (3, –1) points (BCP), brown dots are (3, –3) points (nucleus/NNA).

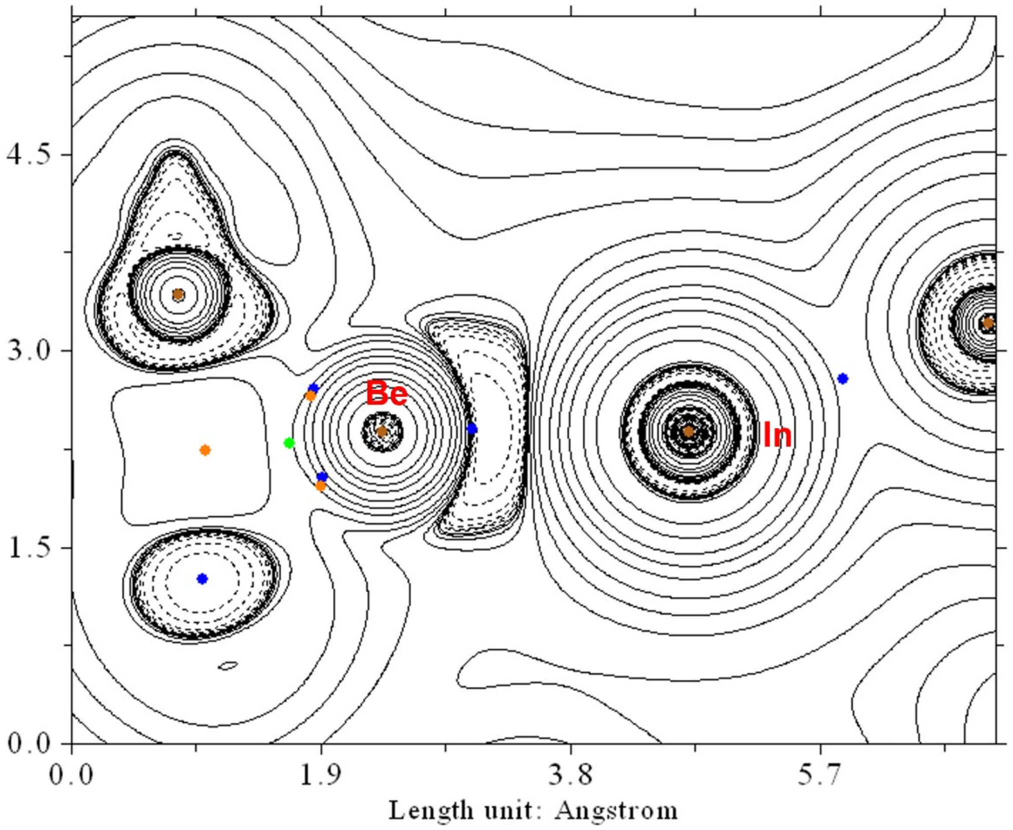


Figure S22: Laplacian of electron density contour plot for **InBe**. Blue dots are (3, –1) points (BCP), brown dots are (3, –3) points (nucleus/NNA).

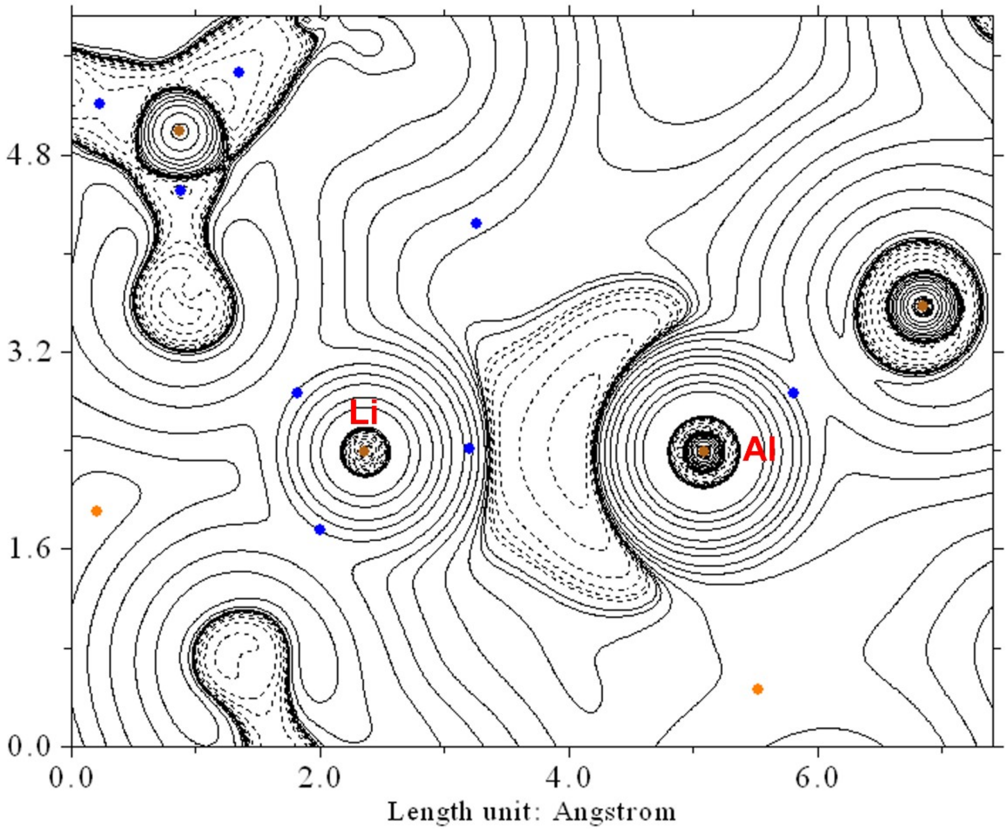


Figure S23: Laplacian of electron density contour plot for **AlLi**. Blue dots are (3, –1) points (BCP), brown dots are (3, –3) points (nucleus/NNA).

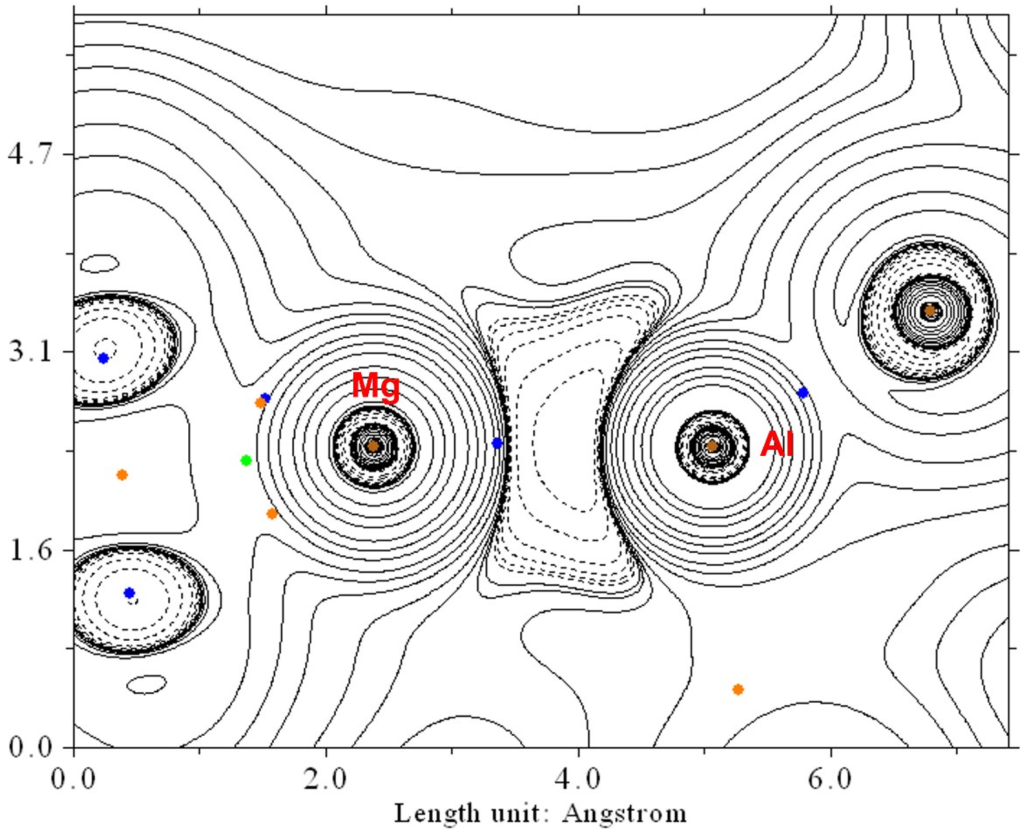


Figure S24: Laplacian of electron density contour plot for **AlMg**. Blue dots are (3, –1) points (BCP), brown dots are (3, –3) points (nucleus/NNA).

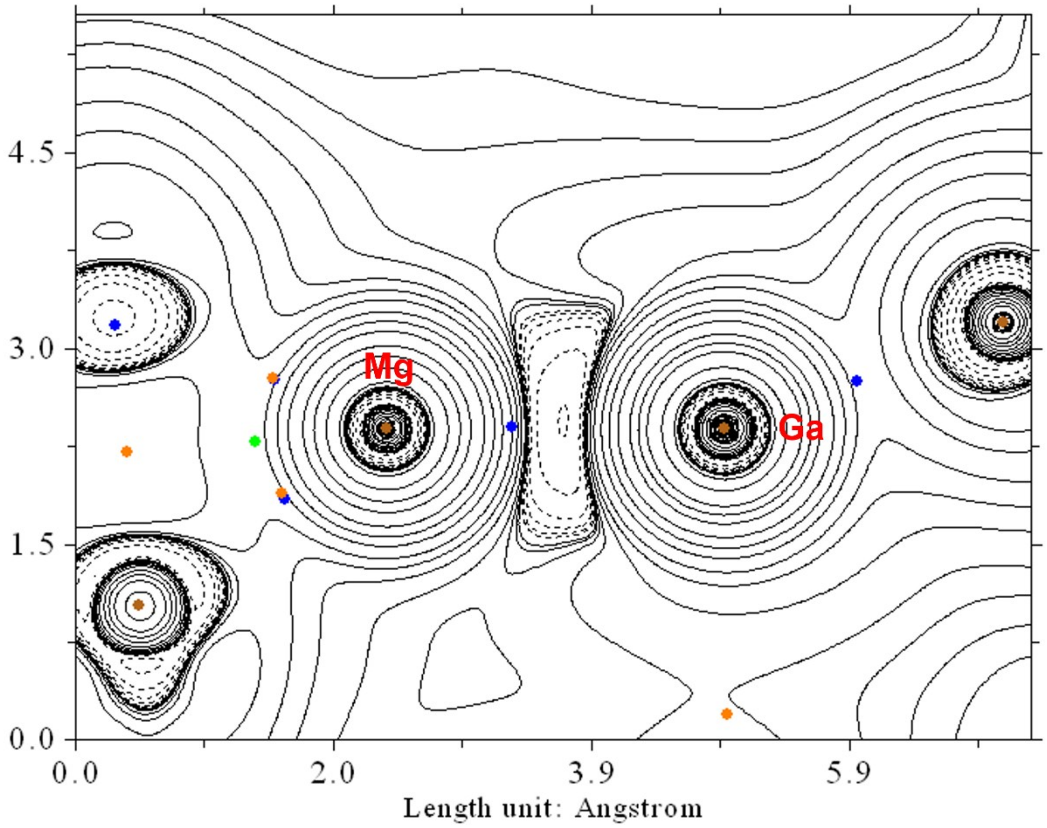


Figure S25: Laplacian of electron density contour plot for **GaMg**. Blue dots are (3, –1) points (BCP), brown dots are (3, –3) points (nucleus/NNA).

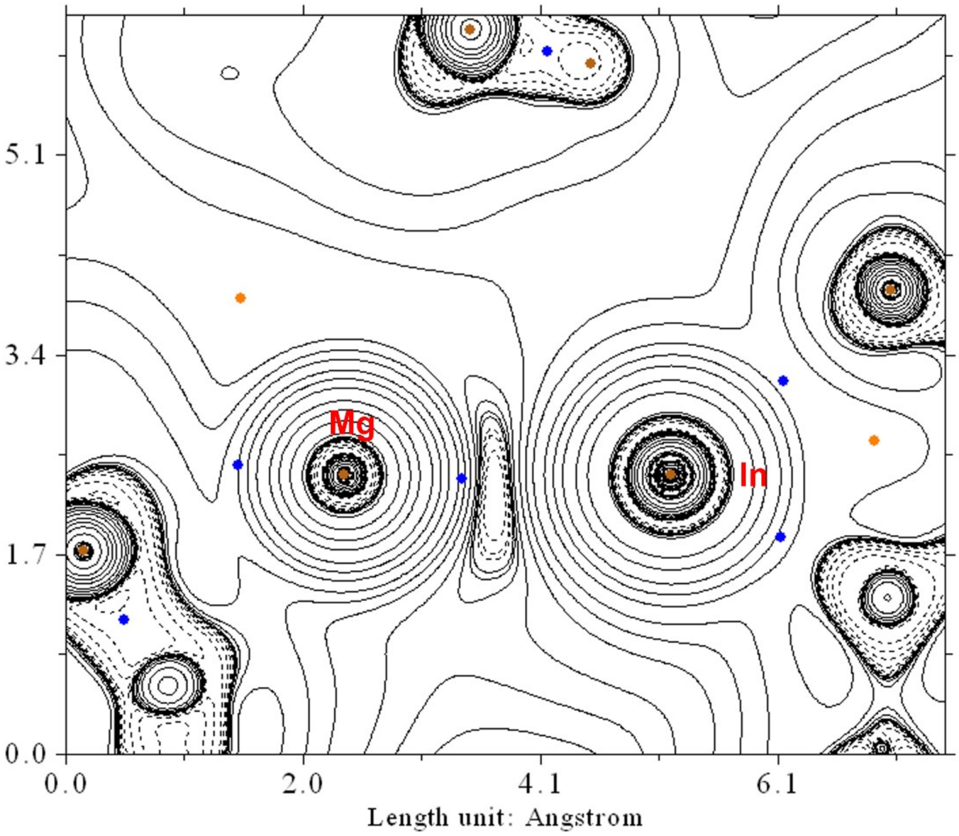


Figure S26: Laplacian of electron density contour plot for **InMg**. Blue dots are (3, –1) points (BCP), brown dots are (3, –3) points (nucleus/NNA).

*Energy Decomposition Analysis with Natural Orbitals for Chemical Valence*

Table S6: EDA-NOCV data for complexes and their respective fragments – homolytic cleavage of metal-metal bond. Shaded boxes represent smallest $|E_{orb}|$.

| | | Pair $\alpha_1$ | | | Pair $\beta_1$ | | |
|---|---|---|---|---|---|---|---|
| | $E_{orb}$ / kcal mol$^{-1}$ | Energy / kcal mol$^{-1}$ | Eigenvalue | % of total | Energy / kcal mol$^{-1}$ | Eigenvalue | % of total |
| **AlBe** | -79.95 | -29.84 | 0.32061 | 45.6 | -43.12 | 0.38232 | 54.4 |
| **GaBe** | -88.07 | -26.15 | 0.2912 | 40.4 | -53.4 | 0.42909 | 59.6 |
| **InBe** | -98.77 | -24.65 | 0.29595 | 39.6 | -65.78 | 0.45097 | 60.4 |
| **AlMg** | -74.94 | -23.55 | 0.31964 | 40.2 | -47.59 | 0.47472 | 59.8 |
| **GaMg** | -81.43 | -19.67 | 0.29049 | 35.9 | -57.14 | 0.5195 | 64.1 |
| **InMg** | -125.44 | -17.24 | 0.28499 | 29.7 | -92.94 | 0.67384 | 70.3 |
| **AlLi** | -86.65 | -9.95 | 0.1968 | 25.1 | -66.9 | 0.58592 | 74.9 |

Table S7: EDA-NOCV data for complexes and their respective fragments – donor-acceptor fragmentation of metal-metal bond. Shaded boxes represent smallest $|E_{orb}|$.

| | M→Tr | | | Tr→M | | |
|---|---|---|---|---|---|---|
| | $E_{orb}$ / kcal mol$^{-1}$ | Pair 1 | Pair 2 | $E_{orb}$ / kcal mol$^{-1}$ | Pair 1 | Pair 2 |
| **AlBe** | -162.72 | -131.004 | -6.986 | -96.86 | -72.524 | -1.983 |
| **GaBe** | -192.45 | -158.724 | -9.454 | -90.18 | -65.34 | -2.496 |
| **InBe** | -221.74 | -188.347 | -11.843 | -82.77 | -61.458 | -2.422 |
| **AlMg** | -125.90 | -107.679 | -3.458 | -75.38 | -50.688 | -2.134 |
| **GaMg** | -147.65 | -129.448 | -3.985 | -71.04 | -45.074 | -3.156 |
| **InMg** | -203.80 | -185.361 | -4.848 | -76.67 | -40.518 | -8.868 |
| **AlLi** | -212.87 | -188.287 | -3.386 | -31.03 | -14.866 | -0.88 |

*Singlet-Triplet Gap Calculations*

Table S8: Calculated S-T gaps for complexes relevant to this study.

| | ΔG / kcal mol$^{-1}$ | ΔE / kcal mol$^{-1}$ |
|---|---|---|
| **AlBe** | -52.0755 | -54.7354 |
| **GaBe** | -50.5548 | -53.8934 |
| **InBe** | -38.3601 | -40.5165 |
| **AlMg** | -50.6708 | -51.7464 |
| **GaMg** | -45.5822 | -48.2929 |
| **InMg** | -34.3381 | -35.8057 |

*Miscellaneous Charge Distribution Data*

Table S9: Calculated charges for Tr and Ae atoms of complexes relevant to this study, as assessed by various computational techniques.

| | Tr | | | Ae | | |
|---|---|---|---|---|---|---|
| | **CHELPG** | **VDD** | **ADCH** | **CHELPG** | **VDD** | **ADCH** |
| **AlBe** | 0.0281 | 0.2442 | 0.188704 | 0.1205 | -0.0652 | -0.14287 |
| **GaBe** | 0.1811 | 0.2422 | 0.224769 | 0.1009 | -0.0177 | -0.10394 |
| **InBe** | 0.4159 | 0.2514 | 0.297786 | 0.0176 | -0.0059 | -0.10023 |
| **AlMg** | -0.0719 | 0.1682 | 0.000945 | 0.2563 | 0.2504 | 0.202162 |
| **GaMg** | 0.1020 | 0.1608 | 0.069526 | 0.2803 | 0.3182 | 0.25552 |
| **InMg** | 0.1686 | 0.1366 | 0.093475 | 0.2256 | 0.3693 | 0.308523 |

*Coordinates of Optimised Structures*

Table S10: xyz-Coordinates of selected optimized structures

**AlBe**

| | | | |
|---|---|---|---|
| Al | 5.27577 | 5.11221 | 8.36417 |
| O | 5.85138 | 6.86350 | 9.28479 |
| N | 4.13913 | 5.10884 | 9.90275 |
| N | 7.16566 | 5.03953 | 8.10861 |
| Be | 4.26948 | 3.92151 | 6.68119 |
| C | 5.21144 | 7.09564 | 10.51355 |
| C | 7.24432 | 7.01185 | 9.37814 |
| C | 2.99963 | 4.26065 | 9.97304 |
| C | 4.25274 | 6.13749 | 10.81774 |
| C | 7.74531 | 4.31636 | 7.02521 |
| C | 7.94768 | 6.01598 | 8.70523 |
| C | 2.79587 | 3.60212 | 5.48565 |
| C | 2.79950 | 2.67652 | 6.55980 |
| C | 4.05082 | 2.00746 | 6.54979 |
| C | 4.82092 | 2.51646 | 5.47144 |
| C | 4.04613 | 3.50403 | 4.81402 |
| C | 5.63705 | 8.10809 | 11.34279 |
| C | 7.78745 | 8.01170 | 10.14668 |
| C | 3.16038 | 2.91264 | 10.36245 |
| C | 1.73291 | 4.75180 | 9.59502 |
| C | 3.54888 | 6.32851 | 12.01800 |
| C | 7.99907 | 2.93239 | 7.14690 |
| C | 8.01100 | 4.98582 | 5.81078 |
| C | 9.34150 | 6.12712 | 8.73243 |
| H | 2.00524 | 4.30112 | 5.25274 |
| H | 2.03552 | 2.57833 | 7.32039 |
| H | 4.39138 | 1.30137 | 7.29429 |
| H | 5.85393 | 2.27678 | 5.25582 |
| H | 4.37406 | 4.11768 | 3.98686 |
| C | 4.94194 | 8.24395 | 12.54708 |
| C | 6.82166 | 8.95660 | 10.86994 |
| C | 9.18808 | 8.06792 | 10.18074 |
| C | 2.04805 | 2.07071 | 10.33879 |
| C | 4.50307 | 2.41673 | 10.86798 |
| C | 0.64570 | 3.87556 | 9.59899 |
| C | 1.51824 | 6.19185 | 9.16173 |
| H | 2.77000 | 5.61962 | 12.27378 |
| C | 3.88443 | 7.37889 | 12.87710 |
| C | 8.55237 | 2.25395 | 6.05965 |
| C | 7.67510 | 2.20087 | 8.43688 |
| C | 8.56129 | 4.26445 | 4.74995 |
| C | 7.68211 | 6.45427 | 5.60084 |
| H | 9.92937 | 5.38563 | 8.19927 |
| C | 9.96490 | 7.14808 | 9.46588 |
| H | 5.22957 | 9.02533 | 13.24057 |
| C | 6.30580 | 10.00428 | 9.85392 |
| C | 7.49856 | 9.68226 | 12.03166 |
| H | 9.66722 | 8.83212 | 10.77763 |
| H | 2.15206 | 1.02915 | 10.62576 |
| C | 0.79793 | 2.54431 | 9.95812 |

**AlMg**

| | | | |
|---|---|---|---|
| Al | 2.58107 | 9.44032 | 12.81795 |
| O | 3.13988 | 8.42727 | 11.14485 |
| N | 4.25913 | 8.77094 | 13.39028 |
| N | 0.91590 | 8.65703 | 12.34184 |
| C | 4.36426 | 7.75033 | 11.28121 |
| C | 0.90785 | 7.75361 | 11.28974 |
| C | 4.97822 | 7.98181 | 12.50977 |
| C | 4.77361 | 6.85185 | 10.32802 |
| C | 2.12683 | 7.59674 | 10.63480 |
| C | 2.41120 | 6.67912 | 9.65432 |
| C | -0.14459 | 6.96157 | 10.82135 |
| H | -1.11815 | 7.06344 | 11.29180 |
| C | 5.99814 | 6.21305 | 10.57098 |
| H | 6.36812 | 5.49627 | 9.85063 |
| C | 3.84995 | 6.62520 | 9.12637 |
| C | 4.14659 | 5.30203 | 8.42202 |
| H | 5.17167 | 5.29338 | 8.04029 |
| H | 3.48420 | 5.17380 | 7.56098 |
| H | 4.01588 | 4.44929 | 9.09566 |
| C | 1.33764 | 5.88239 | 9.23161 |
| H | 1.50875 | 5.13904 | 8.46488 |
| C | -0.32390 | 9.23597 | 12.73720 |
| C | -1.00187 | 10.10666 | 11.85459 |
| C | 6.21568 | 7.35781 | 12.69958 |
| H | 6.74594 | 7.52999 | 13.63143 |
| C | 4.92551 | 9.22107 | 14.56739 |
| C | 6.73130 | 6.48173 | 11.73323 |
| C | 0.06353 | 6.02804 | 9.79429 |
| C | -0.43934 | 10.50032 | 10.49923 |
| H | 0.53306 | 10.01395 | 10.37353 |
| C | 5.79949 | 10.32668 | 14.49365 |
| C | 6.06030 | 11.06490 | 13.19326 |
| H | 5.35649 | 10.68305 | 12.44518 |
| C | -1.11814 | 5.15727 | 9.35056 |
| C | 4.04709 | 7.78612 | 8.12167 |
| H | 3.83987 | 8.75291 | 8.58946 |
| H | 3.36931 | 7.66247 | 7.27013 |
| H | 5.07976 | 7.79450 | 7.75671 |
| C | 8.07886 | 5.80600 | 12.01376 |
| C | -0.83620 | 8.98911 | 14.03120 |
| C | -2.22004 | 10.65627 | 12.25857 |
| H | -2.75620 | 11.31763 | 11.58262 |
| C | 4.69136 | 8.56810 | 15.79636 |
| C | -0.09344 | 8.07603 | 14.99161 |
| H | 0.97301 | 8.34370 | 14.93135 |
| C | -1.34182 | 10.03572 | 9.34848 |
| H | -2.32280 | 10.52269 | 9.39265 |
| H | -1.49434 | 8.95371 | 9.38321 |
| H | -0.88718 | 10.28588 | 8.38332 |
| C | 6.45152 | 10.74209 | 15.65624 |

| | | | |
|---|---|---|---|
| H | 5.28468 | 2.90824 | 10.27440 |
| C | 4.69870 | 0.90642 | 10.73666 |
| C | 4.71560 | 2.85116 | 12.32733 |
| H | -0.33501 | 4.24000 | 9.30330 |
| H | 2.46406 | 6.72900 | 9.27464 |
| C | 1.12878 | 6.26959 | 7.67994 |
| C | 0.47984 | 6.89924 | 10.04233 |
| C | 3.13133 | 7.60349 | 14.19391 |
| H | 8.75881 | 1.19154 | 6.13401 |
| C | 8.83886 | 2.91163 | 4.86980 |
| H | 6.68648 | 2.55582 | 8.76301 |
| C | 7.58065 | 0.68292 | 8.27405 |
| C | 8.67859 | 2.54935 | 9.54665 |
| H | 8.76736 | 4.77093 | 3.81040 |
| H | 7.31539 | 6.86528 | 6.54531 |
| C | 6.55542 | 6.61667 | 4.57094 |
| C | 8.92034 | 7.26653 | 5.20153 |
| C | 11.49748 | 7.19763 | 9.48670 |
| H | 5.58711 | 10.67307 | 10.33969 |
| H | 5.80892 | 9.52289 | 9.00649 |
| H | 7.14265 | 10.59822 | 9.47081 |
| H | 8.32762 | 10.29626 | 11.66781 |
| H | 7.88354 | 8.97827 | 12.77610 |
| H | 6.79222 | 10.35901 | 12.52140 |
| H | -0.05711 | 1.87410 | 9.94648 |
| H | 5.72690 | 0.64427 | 11.00575 |
| H | 4.51318 | 0.55716 | 9.71504 |
| H | 4.03928 | 0.35060 | 11.41251 |
| H | 3.93670 | 2.41867 | 12.96679 |
| H | 4.67737 | 3.93865 | 12.42912 |
| H | 5.69057 | 2.50778 | 12.69203 |
| H | 1.91377 | 5.82689 | 7.05931 |
| H | 0.99378 | 7.31311 | 7.37298 |
| H | 0.19173 | 5.73550 | 7.48319 |
| H | -0.51318 | 6.44853 | 9.93470 |
| H | 0.39989 | 7.95491 | 9.76032 |
| H | 0.76448 | 6.85038 | 11.09807 |
| C | 2.04017 | 6.55281 | 14.43152 |
| C | 2.46158 | 8.99080 | 14.15947 |
| C | 4.11892 | 7.53895 | 15.37450 |
| H | 9.27022 | 2.36496 | 4.03591 |
| H | 8.55732 | 0.23909 | 8.05014 |
| H | 6.88660 | 0.39889 | 7.47509 |
| H | 7.22966 | 0.23033 | 9.20587 |
| H | 8.40868 | 2.04202 | 10.48058 |
| H | 8.71080 | 3.62444 | 9.73868 |
| H | 9.68502 | 2.22175 | 9.25995 |
| H | 6.84570 | 6.21397 | 3.59370 |
| H | 6.30652 | 7.67601 | 4.43970 |
| H | 5.65543 | 6.08982 | 4.90540 |
| H | 9.31079 | 6.95280 | 4.22683 |
| H | 9.71692 | 7.15090 | 5.94266 |
| H | 8.66944 | 8.33087 | 5.13289 |
| C | 12.04017 | 5.89320 | 10.10164 |
| C | 12.02399 | 7.34184 | 8.04646 |
| C | 12.03802 | 8.37335 | 10.30964 |
| H | 1.53418 | 6.75980 | 15.38051 |
| H | 2.45680 | 5.54132 | 14.48929 |
| H | 1.28161 | 6.57029 | 13.64152 |
| H | 1.90264 | 9.16730 | 15.08612 |
| H | 1.76449 | 9.05983 | 13.31708 |
| H | 3.19923 | 9.79187 | 14.05107 |
| H | 4.88926 | 8.31256 | 15.30526 |
| H | 4.62084 | 6.56585 | 15.40478 |
| H | 3.58533 | 7.68185 | 16.32148 |
| H | 11.74169 | 5.01706 | 9.51816 |
| H | 11.66271 | 5.76165 | 11.12153 |
| H | 13.13570 | 5.91762 | 10.13843 |
| H | 13.11892 | 7.39817 | 8.04630 |
| H | 11.63196 | 8.25333 | 7.58184 |
| H | 11.73314 | 6.49212 | 7.42123 |
| H | 13.13270 | 8.36425 | 10.27930 |
| H | 11.73635 | 8.30729 | 11.36063 |
| H | 11.70218 | 9.33647 | 9.90976 |

## GaBe

| | | | |
|---|---|---|---|
| Ga | 5.22916 | 4.98815 | 8.27956 |
| O | 5.84365 | 6.94505 | 9.26553 |
| N | 4.12647 | 5.12504 | 9.90434 |
| N | 7.18150 | 5.05038 | 8.10554 |
| Be | 4.26908 | 3.78138 | 6.68592 |
| C | 5.21215 | 7.13015 | 10.49397 |
| C | 7.22839 | 7.04909 | 9.36715 |
| C | 2.98500 | 4.28709 | 9.96960 |
| C | 4.25973 | 6.16300 | 10.80097 |
| C | 7.75792 | 4.33520 | 7.02214 |
| C | 7.93384 | 6.04390 | 8.70521 |
| C | 2.78714 | 3.49481 | 5.51940 |
| C | 2.80405 | 2.55643 | 6.58319 |

| | | | |
|---|---|---|---|
| H | 7.13357 | 11.58733 | 15.61425 |
| C | 8.52698 | 4.88975 | 10.86869 |
| H | 7.82505 | 4.06369 | 10.71105 |
| H | 9.50131 | 4.45348 | 11.11268 |
| H | 8.63471 | 5.44124 | 9.92830 |
| C | -0.20276 | 12.01577 | 10.42208 |
| H | 0.26535 | 12.28080 | 9.46742 |
| H | 0.45384 | 12.35334 | 11.23172 |
| H | -1.14237 | 12.57325 | 10.50176 |
| C | -2.04721 | 9.57919 | 14.39564 |
| H | -2.45731 | 9.40179 | 15.38380 |
| C | 6.24093 | 10.09683 | 16.86532 |
| H | 6.75701 | 10.43321 | 17.76022 |
| C | -0.75043 | 4.20922 | 8.20257 |
| H | -0.41305 | 4.75788 | 7.31628 |
| H | -1.63162 | 3.62508 | 7.91714 |
| H | 0.03354 | 3.50242 | 8.49518 |
| C | -2.74612 | 10.39298 | 13.51329 |
| H | -3.69290 | 10.83344 | 13.81315 |
| C | 5.36261 | 9.02354 | 16.93219 |
| H | 5.19911 | 8.53279 | 17.88656 |
| C | -2.27370 | 6.05721 | 8.87475 |
| H | -2.63754 | 6.70800 | 9.67549 |
| H | -3.11592 | 5.44372 | 8.53397 |
| H | -1.95243 | 6.69285 | 8.04223 |
| C | 3.77196 | 7.36217 | 15.88116 |
| H | 3.01564 | 7.46690 | 15.09277 |
| C | 5.81431 | 12.57266 | 13.33509 |
| H | 4.82086 | 12.77588 | 13.75073 |
| H | 5.88412 | 13.06318 | 12.35783 |
| H | 6.55234 | 13.04262 | 13.99437 |
| C | -1.59185 | 4.30402 | 10.54316 |
| H | -0.77801 | 3.66840 | 10.90832 |
| H | -2.42551 | 3.65890 | 10.24146 |
| H | -1.93168 | 4.92657 | 11.37664 |
| C | 7.96450 | 4.95475 | 13.29335 |
| H | 7.72079 | 5.56787 | 14.16633 |
| H | 8.91316 | 4.44350 | 13.49513 |
| H | 7.18010 | 4.19770 | 13.18496 |
| C | 9.15932 | 6.88556 | 12.21435 |
| H | 9.24789 | 7.51498 | 11.32201 |
| H | 10.13209 | 6.41723 | 12.40472 |
| H | 8.92863 | 7.53548 | 13.06387 |
| C | 7.48204 | 10.79864 | 12.67662 |
| H | 8.23024 | 11.15573 | 13.39404 |
| H | 7.64906 | 11.31904 | 11.72664 |
| H | 7.64718 | 9.72992 | 12.51328 |
| C | -0.51963 | 8.24526 | 16.45031 |
| H | -0.45283 | 9.28979 | 16.77301 |
| H | 0.12740 | 7.64717 | 17.09766 |
| H | -1.54678 | 7.89957 | 16.61292 |
| C | -0.22357 | 6.60483 | 14.56919 |
| H | -1.27552 | 6.29710 | 14.60106 |
| H | 0.34084 | 5.95895 | 15.25197 |
| H | 0.14991 | 6.44012 | 13.55541 |
| C | 4.53972 | 6.06096 | 15.59660 |
| H | 5.35328 | 5.93396 | 16.32132 |
| H | 4.97309 | 6.06019 | 14.59299 |
| H | 3.86992 | 5.19723 | 15.67939 |
| C | 3.04059 | 7.25661 | 17.22189 |
| H | 2.28129 | 6.46931 | 17.16744 |
| H | 2.54754 | 8.19734 | 17.48883 |
| H | 3.72169 | 6.98616 | 18.03638 |
| Mg | 1.88601 | 11.77256 | 13.94252 |
| C | 0.68813 | 12.63515 | 15.77852 |
| H | 0.28342 | 11.98531 | 16.54384 |
| C | 1.97443 | 13.23750 | 15.78965 |
| H | 2.72343 | 13.12847 | 16.56388 |
| C | 2.09939 | 14.02211 | 14.61312 |
| H | 2.95220 | 14.63148 | 14.34165 |
| C | 0.01709 | 13.04663 | 14.59794 |
| H | -0.97938 | 12.74761 | 14.29684 |
| C | 0.88889 | 13.90395 | 13.87672 |
| H | 0.66047 | 14.40429 | 12.94416 |

## GaMg

| | | | |
|---|---|---|---|
| Ga | 2.50204 | 9.47030 | 12.93000 |
| O | 3.15818 | 8.33444 | 11.07030 |
| N | 4.28826 | 8.79306 | 13.32870 |
| N | 0.88166 | 8.60769 | 12.24196 |
| C | 4.34514 | 7.64295 | 11.27466 |
| C | 0.90085 | 7.74217 | 11.16561 |
| C | 4.96851 | 7.93429 | 12.49020 |
| C | 4.76386 | 6.69543 | 10.37098 |
| C | 2.14095 | 7.54117 | 10.55602 |
| C | 2.41637 | 6.58932 | 9.60205 |
| C | -0.16829 | 7.00761 | 10.64141 |
| H | -1.15466 | 7.15488 | 11.07166 |
| C | 5.97454 | 6.04938 | 10.65909 |

| | | | |
|---|---|---|---|
| C | 4.05487 | 1.88542 | 6.54794 |
| C | 4.81168 | 2.40670 | 5.46506 |
| C | 4.02878 | 3.40359 | 4.82946 |
| C | 5.63720 | 8.12962 | 11.34378 |
| C | 7.78721 | 8.03682 | 10.14565 |
| C | 3.13877 | 2.93515 | 10.35493 |
| C | 1.72293 | 4.77926 | 9.57405 |
| C | 3.56302 | 6.34236 | 12.00906 |
| C | 8.00604 | 2.94829 | 7.14141 |
| C | 8.01079 | 5.00140 | 5.80219 |
| C | 9.32943 | 6.14696 | 8.74187 |
| H | 1.99477 | 4.19874 | 5.30825 |
| H | 2.05101 | 2.45342 | 7.35426 |
| H | 4.40628 | 1.17216 | 7.28028 |
| H | 5.84168 | 2.16893 | 5.23292 |
| H | 4.34773 | 4.02818 | 4.00693 |
| C | 4.95569 | 8.24910 | 12.55630 |
| C | 6.82287 | 8.98042 | 10.87441 |
| C | 9.18659 | 8.08137 | 10.19409 |
| C | 2.02436 | 2.09651 | 10.31955 |
| C | 4.47719 | 2.43412 | 10.86768 |
| C | 0.63388 | 3.90565 | 9.56597 |
| C | 1.52018 | 6.21986 | 9.13801 |
| H | 2.78816 | 5.62780 | 12.26191 |
| C | 3.89975 | 7.38057 | 12.88093 |
| C | 8.54810 | 2.26808 | 6.04973 |
| C | 7.69704 | 2.21935 | 8.43732 |
| C | 8.55358 | 4.27878 | 4.73868 |
| C | 7.67001 | 6.46687 | 5.59419 |
| H | 9.91414 | 5.39928 | 8.21354 |
| C | 9.96003 | 7.15963 | 9.47849 |
| H | 5.25191 | 9.01765 | 13.26069 |
| C | 6.30788 | 10.03571 | 9.86560 |
| C | 7.50125 | 9.69921 | 12.03940 |
| H | 9.66806 | 8.83617 | 10.80130 |
| H | 2.12287 | 1.05433 | 10.60672 |
| C | 0.77881 | 2.57395 | 9.92778 |
| H | 5.26335 | 2.92490 | 10.27964 |
| C | 4.66937 | 0.92331 | 10.73538 |
| C | 4.68451 | 2.86696 | 12.32809 |
| H | -0.34286 | 4.27250 | 9.25992 |
| H | 2.47046 | 6.74877 | 9.25179 |
| C | 1.13164 | 6.30046 | 7.65582 |
| C | 0.48625 | 6.93537 | 10.01725 |
| C | 3.15538 | 7.58440 | 14.20605 |
| H | 8.75366 | 1.20525 | 6.12299 |
| C | 8.82926 | 2.92513 | 4.85784 |
| H | 6.71199 | 2.57100 | 8.77800 |
| C | 7.60457 | 0.70053 | 8.28089 |
| C | 8.71210 | 2.57407 | 9.53459 |
| H | 8.75388 | 4.78412 | 3.79713 |
| H | 7.28833 | 6.86942 | 6.53627 |
| C | 6.55369 | 6.62241 | 4.55152 |
| C | 8.90489 | 7.29186 | 5.21142 |
| C | 11.49289 | 7.19651 | 9.50370 |
| H | 5.59082 | 10.70243 | 10.35685 |
| H | 5.80987 | 9.56075 | 9.01551 |
| H | 7.14561 | 10.63125 | 9.48658 |
| H | 8.33050 | 10.31489 | 11.67844 |
| H | 7.88668 | 8.99095 | 12.77969 |
| H | 6.79552 | 10.37342 | 12.53380 |
| H | -0.07822 | 1.90637 | 9.90895 |
| H | 5.69681 | 0.65851 | 11.00490 |
| H | 4.48423 | 0.57462 | 9.71341 |
| H | 4.00787 | 0.36813 | 11.40979 |
| H | 3.90009 | 2.43863 | 12.96364 |
| H | 4.65242 | 3.95464 | 12.42878 |
| H | 5.65584 | 2.51810 | 12.69716 |
| H | 1.91167 | 5.85282 | 7.03181 |
| H | 1.00516 | 7.34495 | 7.34897 |
| H | 0.19046 | 5.77416 | 7.45795 |
| H | -0.50937 | 6.48987 | 9.91094 |
| H | 0.41278 | 7.99114 | 9.73404 |
| H | 0.77200 | 6.88621 | 11.07259 |
| C | 2.05872 | 6.53640 | 14.42976 |
| C | 2.49461 | 8.97633 | 14.20436 |
| C | 4.14993 | 7.49028 | 15.37890 |
| H | 9.25488 | 2.37723 | 4.02178 |
| H | 8.58042 | 0.25764 | 8.05163 |
| H | 6.90583 | 0.41089 | 7.48795 |
| H | 7.26085 | 0.25147 | 9.21726 |
| H | 8.45788 | 2.06280 | 10.47067 |
| H | 8.73711 | 3.64899 | 9.72790 |
| H | 9.71720 | 2.25509 | 9.23390 |
| H | 6.86410 | 6.24210 | 3.57156 |
| H | 6.28444 | 7.67823 | 4.43398 |
| H | 5.65858 | 6.07238 | 4.86219 |
| H | 9.31404 | 6.97752 | 4.24451 |
| H | 9.68979 | 7.18731 | 5.96648 |
| H | 8.64398 | 8.35323 | 5.13490 |
| H | 6.34227 | 5.29387 | 9.97788 |
| C | 3.87453 | 6.45520 | 9.14448 |
| C | 4.14393 | 5.09335 | 8.50491 |
| H | 5.18191 | 5.02726 | 8.16593 |
| H | 3.51174 | 4.95488 | 7.62309 |
| H | 3.95097 | 4.27553 | 9.20650 |
| C | 1.32838 | 5.83829 | 9.13595 |
| H | 1.49864 | 5.07465 | 8.38897 |
| C | -0.34736 | 9.17494 | 12.66620 |
| C | -0.97379 | 10.16108 | 11.87171 |
| C | 6.19317 | 7.30090 | 12.72363 |
| H | 6.72414 | 7.52659 | 13.64413 |
| C | 4.97925 | 9.41193 | 14.40313 |
| C | 6.70341 | 6.36521 | 11.81169 |
| C | 0.03677 | 6.05928 | 9.62880 |
| C | -0.38469 | 10.63309 | 10.55470 |
| H | 0.55213 | 10.09370 | 10.38359 |
| C | 5.79706 | 10.53624 | 14.14667 |
| C | 6.01624 | 11.06087 | 12.73979 |
| H | 5.38413 | 10.47813 | 12.06164 |
| C | -1.16828 | 5.25076 | 9.13388 |
| C | 4.16408 | 7.56675 | 8.10652 |
| H | 3.97828 | 8.55732 | 8.53176 |
| H | 3.51776 | 7.43941 | 7.23121 |
| H | 5.21086 | 7.51890 | 7.78693 |
| C | 8.04294 | 5.69182 | 12.13299 |
| C | -0.88670 | 8.82046 | 13.92597 |
| C | -2.15920 | 10.73982 | 12.32857 |
| H | -2.65335 | 11.49378 | 11.72095 |
| C | 4.78659 | 8.95496 | 15.72307 |
| C | -0.22293 | 7.75206 | 14.78093 |
| H | 0.86228 | 7.93496 | 14.75736 |
| C | -1.31696 | 10.32274 | 9.37593 |
| H | -2.26513 | 10.86532 | 9.46492 |
| H | -1.53802 | 9.25246 | 9.32580 |
| H | -0.84826 | 10.61899 | 8.43089 |
| C | 6.41548 | 11.17506 | 15.22132 |
| H | 7.04769 | 12.04021 | 15.03800 |
| C | 8.48063 | 4.70217 | 11.04647 |
| H | 7.76813 | 3.87732 | 10.93621 |
| H | 9.44881 | 4.26912 | 11.31915 |
| H | 8.59755 | 5.19371 | 10.07447 |
| C | -0.04683 | 12.13026 | 10.60594 |
| H | 0.43584 | 12.44495 | 9.67397 |
| H | 0.63559 | 12.35184 | 11.43520 |
| H | -0.94569 | 12.74180 | 10.74150 |
| C | -2.06608 | 9.43965 | 14.34480 |
| H | -2.49757 | 9.18284 | 15.30612 |
| C | 6.23368 | 10.72849 | 16.52370 |
| H | 6.72364 | 11.23858 | 17.34839 |
| C | -0.80321 | 4.29256 | 7.99355 |
| H | -0.40029 | 4.83060 | 7.12840 |
| H | -1.70074 | 3.75729 | 7.66591 |
| H | -0.06964 | 3.54381 | 8.31124 |
| C | -2.70603 | 10.38338 | 13.55138 |
| H | -3.62684 | 10.84719 | 13.89411 |
| C | 5.42469 | 9.62846 | 16.76744 |
| H | 5.29122 | 9.28142 | 17.78876 |
| C | -2.25987 | 6.20864 | 8.62137 |
| H | -2.62140 | 6.87138 | 9.41318 |
| H | -3.11742 | 5.63826 | 8.24556 |
| H | -1.87649 | 6.83261 | 7.80652 |
| C | 3.95123 | 7.72602 | 16.02562 |
| H | 3.43191 | 7.44221 | 15.10284 |
| C | 5.59800 | 12.53168 | 12.61094 |
| H | 4.55681 | 12.67380 | 12.92354 |
| H | 5.69066 | 12.86425 | 11.57115 |
| H | 6.22456 | 13.18583 | 13.22707 |
| C | -1.72970 | 4.41581 | 10.30155 |
| H | -0.96179 | 3.74214 | 10.69699 |
| H | -2.57975 | 3.81108 | 9.96361 |
| H | -2.07356 | 5.05135 | 11.12374 |
| C | 7.92327 | 4.92059 | 13.46186 |
| H | 7.69892 | 5.59011 | 14.29785 |
| H | 8.86392 | 4.40491 | 13.68860 |
| H | 7.12383 | 4.17401 | 13.40425 |
| C | 9.13519 | 6.77002 | 12.26740 |
| H | 9.23223 | 7.34023 | 11.33695 |
| H | 10.10281 | 6.30559 | 12.49123 |
| H | 8.90672 | 7.47510 | 13.07236 |
| C | 7.47321 | 10.86453 | 12.29720 |
| H | 8.15877 | 11.44018 | 12.92996 |
| H | 7.60726 | 11.19891 | 11.26233 |
| H | 7.75839 | 9.80951 | 12.35473 |
| C | -0.66292 | 7.77848 | 16.24501 |
| H | -0.53160 | 8.76832 | 16.69597 |
| H | -0.07033 | 7.06220 | 16.82106 |
| H | -1.71374 | 7.48761 | 16.35467 |
| C | -0.45807 | 6.35225 | 14.19084 |
| H | -1.53215 | 6.13288 | 14.15974 |

| | | | |
|---|---|---|---|
| C | 12.02497 | 5.88616 | 10.11527 |
| C | 12.02412 | 7.34173 | 8.06524 |
| C | 12.04007 | 8.36541 | 10.33194 |
| H | 1.55686 | 6.73025 | 15.38380 |
| H | 2.46962 | 5.52165 | 14.46933 |
| H | 1.29810 | 6.57127 | 13.64220 |
| H | 1.94254 | 9.13781 | 15.13795 |
| H | 1.79307 | 9.06774 | 13.36780 |
| H | 3.23711 | 9.77438 | 14.10763 |
| H | 4.92513 | 8.25968 | 15.31789 |
| H | 4.64526 | 6.51336 | 15.38658 |
| H | 3.62420 | 7.61887 | 16.33245 |
| H | 11.72127 | 5.01431 | 9.52798 |
| H | 11.64374 | 5.75386 | 11.13367 |
| H | 13.12066 | 5.90207 | 10.15490 |
| H | 13.11961 | 7.38844 | 8.06708 |
| H | 11.64081 | 8.25846 | 7.60363 |
| H | 11.72653 | 6.49706 | 7.43621 |
| H | 13.13480 | 8.34929 | 10.30372 |
| H | 11.73560 | 8.29748 | 11.38199 |
| H | 11.71079 | 9.33204 | 9.93514 |

| | | | |
|---|---|---|---|
| H | 0.02759 | 5.59038 | 14.81131 |
| H | -0.06246 | 6.26889 | 13.17630 |
| C | 4.85016 | 6.54699 | 16.42586 |
| H | 5.40506 | 6.77018 | 17.34474 |
| H | 5.57450 | 6.32477 | 15.63612 |
| H | 4.24945 | 5.64730 | 16.60044 |
| C | 2.89497 | 7.99666 | 17.10162 |
| H | 2.27040 | 7.10968 | 17.25000 |
| H | 2.24346 | 8.83159 | 16.81771 |
| H | 3.35179 | 8.24154 | 18.06664 |
| Mg | 1.85001 | 11.46663 | 14.42914 |
| C | 0.92304 | 12.05345 | 16.49300 |
| H | 0.57925 | 11.31313 | 17.20418 |
| C | 2.23046 | 12.60803 | 16.43398 |
| H | 3.06245 | 12.35314 | 17.07870 |
| C | 2.25487 | 13.55758 | 15.37909 |
| H | 3.10342 | 14.16669 | 15.09377 |
| C | 0.13872 | 12.65976 | 15.47555 |
| H | -0.90050 | 12.44508 | 15.25925 |
| C | 0.96158 | 13.58964 | 14.78588 |
| H | 0.65048 | 14.22848 | 13.96882 |

## InBe

| | | | |
|---|---|---|---|
| In | 5.21378 | 9.61583 | 4.73999 |
| O | 4.57896 | 8.21774 | 6.63833 |
| N | 3.24949 | 8.78748 | 4.45213 |
| N | 6.94498 | 8.60243 | 5.56835 |
| C | 6.34628 | 13.32868 | 3.57753 |
| H | 6.41432 | 13.92894 | 4.47412 |
| C | 5.18541 | 13.15905 | 2.77262 |
| H | 4.21465 | 13.59955 | 2.95209 |
| C | 5.49183 | 12.21827 | 1.75492 |
| H | 4.78793 | 11.81178 | 1.04125 |
| C | 6.84148 | 11.80866 | 1.93089 |
| H | 7.35262 | 11.03975 | 1.36884 |
| C | 7.36995 | 12.49193 | 3.05726 |
| H | 8.34279 | 12.31878 | 3.49968 |
| C | 2.76396 | 6.66593 | 1.06512 |
| H | 2.34015 | 6.97635 | 0.10321 |
| H | 1.94011 | 6.34107 | 1.70842 |
| H | 3.41518 | 5.80374 | 0.88295 |
| C | 3.55438 | 7.80528 | 1.72309 |
| H | 3.97854 | 7.41586 | 2.65604 |
| C | 4.71310 | 8.25527 | 0.82458 |
| H | 5.37130 | 7.41159 | 0.59101 |
| H | 5.31315 | 9.03190 | 1.31441 |
| H | 4.34576 | 8.66529 | -0.12344 |
| C | 2.64774 | 8.96553 | 2.08595 |
| C | 1.95175 | 9.63683 | 1.07761 |
| H | 2.08330 | 9.33164 | 0.04214 |
| C | 2.46918 | 9.37119 | 3.42487 |
| C | 1.57390 | 10.41959 | 3.73694 |
| C | 0.89439 | 11.05652 | 2.69919 |
| H | 0.20893 | 11.86895 | 2.92842 |
| C | 1.08275 | 10.67651 | 1.37567 |
| H | 0.54586 | 11.18525 | 0.57982 |
| C | 1.36835 | 10.88779 | 5.16567 |
| H | 1.92932 | 10.21712 | 5.82342 |
| C | -0.10504 | 10.81269 | 5.58414 |
| H | -0.72797 | 11.49590 | 4.99588 |
| H | -0.21491 | 11.08564 | 6.63959 |
| H | -0.49237 | 9.79758 | 5.45283 |
| C | 1.92802 | 12.30431 | 5.36189 |
| H | 2.99121 | 12.34332 | 5.09627 |
| H | 1.82313 | 12.62001 | 6.40624 |
| H | 1.39711 | 13.03009 | 4.73459 |
| C | 8.19060 | 9.10729 | 5.13349 |
| C | 8.68821 | 8.73619 | 3.85977 |
| C | 7.97578 | 7.67532 | 3.03674 |
| H | 6.89497 | 7.87519 | 3.09957 |
| C | 8.35962 | 7.67330 | 1.55686 |
| H | 8.23176 | 8.65745 | 1.09268 |
| H | 7.73107 | 6.96085 | 1.01421 |
| H | 9.39966 | 7.36025 | 1.41129 |
| C | 8.20694 | 6.27774 | 3.63478 |
| H | 9.27611 | 6.03442 | 3.61567 |
| H | 7.67317 | 5.51856 | 3.05157 |
| H | 7.86205 | 6.21998 | 4.66936 |
| C | 9.86485 | 9.32939 | 3.39989 |
| H | 10.26137 | 9.05823 | 2.42688 |
| C | 10.55129 | 10.26129 | 4.16994 |
| H | 11.46979 | 10.70538 | 3.79627 |
| C | 10.05812 | 10.62012 | 5.41603 |
| H | 10.59314 | 11.35715 | 6.01019 |
| C | 8.87740 | 10.06494 | 5.91224 |
| C | 8.33259 | 10.54878 | 7.24331 |
| H | 7.45593 | 9.94454 | 7.49385 |
| C | 7.86789 | 12.00879 | 7.13276 |
| H | 8.70603 | 12.67425 | 6.89435 |

## InMg

| | | | |
|---|---|---|---|
| In | 2.60749 | 9.68782 | 13.22697 |
| O | 3.13033 | 8.31103 | 11.25805 |
| N | 4.56821 | 8.84812 | 13.40683 |
| N | 0.78288 | 8.55939 | 12.43086 |
| C | 4.36300 | 7.67151 | 11.34257 |
| C | 0.84980 | 7.71389 | 11.34778 |
| C | 5.11095 | 7.97863 | 12.48871 |
| C | 4.74553 | 6.76469 | 10.37868 |
| C | 2.10394 | 7.52344 | 10.74875 |
| C | 2.36135 | 6.60790 | 9.75044 |
| C | -0.20687 | 6.95099 | 10.82890 |
| H | -1.18946 | 7.07452 | 11.27689 |
| C | 5.99391 | 6.14716 | 10.53440 |
| H | 6.32014 | 5.42855 | 9.79525 |
| C | 3.78967 | 6.54105 | 9.20495 |
| C | 4.06457 | 5.22112 | 8.48437 |
| H | 5.08181 | 5.20821 | 8.08285 |
| H | 3.38767 | 5.10272 | 7.63333 |
| H | 3.94075 | 4.36371 | 9.15363 |
| C | 1.28310 | 5.84347 | 9.28599 |
| H | 1.45926 | 5.10803 | 8.51307 |
| C | -0.38390 | 9.22900 | 12.75833 |
| C | -1.02560 | 10.12485 | 11.84855 |
| C | 6.36263 | 7.35526 | 12.59397 |
| H | 6.97151 | 7.57985 | 13.46452 |
| C | 5.33603 | 9.17982 | 14.55397 |
| C | 6.81147 | 6.44822 | 11.62735 |
| C | -0.00244 | 6.01387 | 9.80958 |
| C | -0.44676 | 10.41823 | 10.47550 |
| H | 0.47075 | 9.83404 | 10.36403 |
| C | 6.26424 | 10.24064 | 14.48922 |
| C | 6.47345 | 11.02047 | 13.20531 |
| H | 5.98652 | 10.46443 | 12.39762 |
| C | -1.18861 | 5.16267 | 9.34000 |
| C | 3.97513 | 7.70529 | 8.20017 |
| H | 3.78389 | 8.66988 | 8.67982 |
| H | 3.27923 | 7.59119 | 7.36160 |
| H | 5.00095 | 7.70905 | 7.81564 |
| C | 8.18683 | 5.79855 | 11.81802 |
| C | -0.86318 | 9.15560 | 14.10594 |
| C | -2.19969 | 10.76681 | 12.24245 |
| H | -2.70498 | 11.42189 | 11.53665 |
| C | 5.14405 | 8.46747 | 15.75565 |
| C | -0.22493 | 8.17743 | 15.08147 |
| H | 0.86618 | 8.23556 | 14.95568 |
| C | -1.40036 | 10.00699 | 9.34788 |
| H | -2.32815 | 10.59022 | 9.37577 |
| H | -1.65566 | 8.94696 | 9.42254 |
| H | -0.92896 | 10.17395 | 8.37337 |
| C | 6.96890 | 10.58692 | 15.64171 |
| H | 7.67781 | 11.41028 | 15.61009 |
| C | 8.56092 | 4.86593 | 10.65986 |
| H | 7.85805 | 4.03083 | 10.56640 |
| H | 9.55510 | 4.44301 | 10.83949 |
| H | 8.59406 | 5.40122 | 9.70450 |
| C | -0.06462 | 11.90136 | 10.35490 |
| H | 0.39073 | 12.09845 | 9.37821 |
| H | 0.65839 | 12.19066 | 11.12763 |
| H | -0.93943 | 12.55337 | 10.45771 |
| C | -2.03098 | 9.85036 | 14.45018 |
| H | -2.41350 | 9.77659 | 15.46303 |
| C | 6.77987 | 9.89612 | 16.83221 |
| H | 7.33953 | 10.17745 | 17.71990 |
| C | -0.81635 | 4.22758 | 8.18292 |
| H | -0.45888 | 4.78634 | 7.31096 |
| H | -1.69966 | 3.65753 | 7.87578 |

| | | | |
|---|---|---|---|
| H | 7.42545 | 12.34624 | 8.07709 |
| H | 7.11559 | 12.11605 | 6.34272 |
| C | 9.34656 | 10.37767 | 8.38067 |
| H | 9.67084 | 9.33552 | 8.45783 |
| H | 8.89587 | 10.66531 | 9.33697 |
| H | 10.23386 | 11.00280 | 8.22978 |
| C | 2.67811 | 7.86774 | 5.29893 |
| C | 1.45205 | 7.21679 | 5.11038 |
| H | 0.87441 | 7.46740 | 4.22501 |
| C | 0.99649 | 6.24602 | 6.01116 |
| C | -0.34294 | 5.55652 | 5.72664 |
| C | -1.45799 | 6.61565 | 5.63767 |
| H | -1.53039 | 7.17996 | 6.57403 |
| H | -2.42506 | 6.13493 | 5.44794 |
| H | -1.27371 | 7.32832 | 4.82815 |
| C | -0.25112 | 4.80083 | 4.38672 |
| H | -0.04791 | 5.48248 | 3.55512 |
| H | -1.19365 | 4.28161 | 4.17553 |
| H | 0.55412 | 4.05895 | 4.41810 |
| C | -0.73030 | 4.54993 | 6.81670 |
| H | -0.00224 | 3.73527 | 6.89649 |
| H | -1.70041 | 4.10429 | 6.57205 |
| H | -0.82189 | 5.03136 | 7.79651 |
| C | 1.77852 | 5.91630 | 7.12218 |
| H | 1.44631 | 5.15017 | 7.80926 |
| C | 2.99687 | 6.56653 | 7.36582 |
| C | 3.90194 | 6.33101 | 8.57872 |
| C | 3.66087 | 4.96094 | 9.21350 |
| H | 3.86718 | 4.15023 | 8.50746 |
| H | 2.62613 | 4.87277 | 9.55681 |
| H | 4.29808 | 4.83063 | 10.09273 |
| C | 3.59664 | 7.42940 | 9.62707 |
| H | 4.25008 | 7.30806 | 10.49812 |
| H | 2.55213 | 7.36075 | 9.95073 |
| H | 3.76313 | 8.42579 | 9.20673 |
| C | 3.38535 | 7.53010 | 6.46262 |
| C | 5.61461 | 7.45948 | 7.17060 |
| C | 5.35442 | 6.49848 | 8.12197 |
| C | 6.87800 | 7.71668 | 6.61621 |
| C | 6.44884 | 5.77703 | 8.61914 |
| H | 6.27803 | 5.01008 | 9.36205 |
| C | 7.94951 | 7.00439 | 7.17458 |
| H | 8.94394 | 7.18921 | 6.77831 |
| C | 7.74582 | 6.03595 | 8.16507 |
| C | 8.95723 | 5.24945 | 8.67945 |
| C | 9.54633 | 4.42151 | 7.52032 |
| H | 10.40344 | 3.83248 | 7.86868 |
| H | 9.88695 | 5.06278 | 6.70106 |
| H | 8.79474 | 3.73383 | 7.11761 |
| C | 10.02946 | 6.22247 | 9.20361 |
| H | 9.62576 | 6.84894 | 10.00662 |
| H | 10.39784 | 6.88198 | 8.41248 |
| H | 10.88660 | 5.66384 | 9.59776 |
| C | 8.59161 | 4.28801 | 9.81698 |
| H | 9.49292 | 3.76880 | 10.16013 |
| H | 7.87570 | 3.52618 | 9.49043 |
| H | 8.16567 | 4.82190 | 10.67365 |
| Be | 5.79757 | 11.51757 | 3.49663 |
| H | -0.04430 | 3.50817 | 8.47597 |
| C | -2.71704 | 10.62238 | 13.52354 |
| H | -3.62651 | 11.14114 | 13.81092 |
| C | 5.87648 | 8.84419 | 16.88333 |
| H | 5.73963 | 8.30291 | 17.81625 |
| C | -2.33446 | 6.07484 | 8.86529 |
| H | -2.70619 | 6.71270 | 9.67296 |
| H | -3.17554 | 5.47081 | 8.50493 |
| H | -2.00108 | 6.72283 | 8.04715 |
| C | 4.18755 | 7.29293 | 15.83940 |
| H | 3.65575 | 7.22837 | 14.88265 |
| C | 5.79948 | 12.39767 | 13.30032 |
| H | 4.73016 | 12.29849 | 13.52247 |
| H | 5.90584 | 12.94816 | 12.35837 |
| H | 6.25120 | 12.99749 | 14.09920 |
| C | -1.68101 | 4.29729 | 10.51644 |
| H | -0.87506 | 3.65154 | 10.88117 |
| H | -2.51716 | 3.66200 | 10.20020 |
| H | -2.02241 | 4.91362 | 11.35421 |
| C | 8.18359 | 4.97276 | 13.11902 |
| H | 7.99954 | 5.60362 | 13.99418 |
| H | 9.15156 | 4.47685 | 13.25911 |
| H | 7.40278 | 4.20510 | 13.08576 |
| C | 9.26427 | 6.89568 | 11.91592 |
| H | 9.26567 | 7.51867 | 11.01473 |
| H | 10.25730 | 6.44413 | 12.02683 |
| H | 9.09542 | 7.54919 | 12.77698 |
| C | 7.95542 | 11.15802 | 12.83759 |
| H | 8.50149 | 11.77481 | 13.55997 |
| H | 8.05714 | 11.63360 | 11.85591 |
| H | 8.43891 | 10.17688 | 12.79258 |
| C | -0.53539 | 8.47543 | 16.54814 |
| H | -0.30444 | 9.51432 | 16.81527 |
| H | 0.06319 | 7.82361 | 17.19137 |
| H | -1.58790 | 8.28803 | 16.78823 |
| C | -0.63440 | 6.74166 | 14.71616 |
| H | -1.72188 | 6.62747 | 14.79592 |
| H | -0.16248 | 6.02559 | 15.39820 |
| H | -0.33098 | 6.49599 | 13.69492 |
| C | 4.94844 | 5.97262 | 16.02603 |
| H | 5.50911 | 5.97101 | 16.96807 |
| H | 5.65650 | 5.81272 | 15.20692 |
| H | 4.25142 | 5.12708 | 16.04470 |
| C | 3.14302 | 7.48535 | 16.94608 |
| H | 2.40448 | 6.67566 | 16.91634 |
| H | 2.61573 | 8.43949 | 16.83186 |
| H | 3.60274 | 7.47824 | 17.94056 |
| Mg | 0.65371 | 11.44485 | 14.13743 |
| C | -0.61837 | 12.87802 | 15.44814 |
| H | -1.52933 | 12.53062 | 15.91727 |
| C | 0.67916 | 12.87943 | 16.03021 |
| H | 0.93064 | 12.52862 | 17.02330 |
| C | 1.57707 | 13.45608 | 15.09772 |
| H | 2.64208 | 13.58765 | 15.24063 |
| C | -0.51508 | 13.44490 | 14.14871 |
| H | -1.33305 | 13.60252 | 13.45751 |
| C | 0.84576 | 13.80302 | 13.93716 |
| H | 1.24603 | 14.27400 | 13.04806 |

**References**


1 M. R. Buchner and M. Müller, *ACS Chem. Heal. Saf.*, 2023, **30**, 36–43.

2 J. Hicks, P. Vasko, J. M. Goicoechea and S. Aldridge, *Nature*, 2018, **557**, 92–95.

3 T. K. Panda, M. T. Gamer and P. W. Roesky, *Organometallics*, 2003, **22**, 877–878.

4 H. Schmidbaur, *Be Organoberyllium Compounds*, Springer Berlin Heidelberg, Berlin, Heidelberg, 1987, vol. 21.

5 E. G. Rochow, Ed., *Inorganic Syntheses*, Wiley, 1960, vol. 6.

6 J. Cosier and A. M. Glazer, *J. Appl. Crystallogr.*, 1986, **19**, 105–107.

7 Agilent Technologies.

8 G. M. Sheldrick, *Acta Crystallogr. Sect. C Struct. Chem.*, 2015, **71**, 3–8.

9 G. M. Sheldrick, *Acta Crystallogr. Sect. A Found. Adv.*, 2015, **71**, 3–8.

10 O. V. Dolomanov, L. J. Bourhis, R. J. Gildea, J. A. K. Howard and H. Puschmann, *J. Appl. Crystallogr.*, 2009, **42**, 339–341.

11 J. K. Buchanan and P. G. Plieger, *Zeitschrift fur Naturforsch. - Sect. B J. Chem. Sci.*, 2020, **75**, 459–472.

12 P. G. Plieger, K. D. John, T. S. Keizer, T. M. McCleskey, A. K. Burrell and R. L. Martin, *J. Am. Chem. Soc.*, 2004, **126**, 14651–14658.

13 J. T. Boronski, L. R. Thomas-Hargreaves, M. A. Ellwanger, A. E. Crumpton, J. Hicks, D. F. Bekiş, S. Aldridge and M. R. Buchner, *J. Am. Chem. Soc.*, 2023, **145**, 4408–4413.

14 F. Neese, *WIREs Comput. Mol. Sci.*, 2012, **2**, 73–78.

15 F. Neese, *WIREs Comput. Mol. Sci.*, DOI:10.1002/wcms.1327.

16 S. Grimme, A. Hansen, S. Ehlert and J.-M. Mewes, *J. Chem. Phys.*, DOI:10.1063/5.0040021.

17 J. W. Furness, A. D. Kaplan, J. Ning, J. P. Perdew and J. Sun, *J. Phys. Chem. Lett.*, 2020, **11**, 8208–8215.

18 E. Caldeweyher, J.-M. Mewes, S. Ehlert and S. Grimme, *Phys. Chem. Chem. Phys.*, 2020, **22**, 8499–8512.

19 H. Kruse and S. Grimme, *J. Chem. Phys.*, DOI:10.1063/1.3700154.

20 V. Barone and M. Cossi, *J. Phys. Chem. A*, 1998, **102**, 1995–2001.

21 N. Mardirossian and M. Head-Gordon, *Phys. Chem. Chem. Phys.*, 2014, **16**, 9904.

22 A. Najibi and L. Goerigk, *J. Comput. Chem.*, 2020, **41**, 2562–2572.

23 F. Weigend and R. Ahlrichs, *Phys. Chem. Chem. Phys.*, 2005, **7**, 3297.

24 F. Weigend, *Phys. Chem. Chem. Phys.*, 2006, **8**, 1057.

25 E. D. Glendening, J. K. Badenhoop, A. E. Reed, J. E. Carpenter, B. J. A., C. M. Morales, P. Karafiloglou, C. R. Landis and F. Weinhold, 2018.

26 T. Lu and F. Chen, *J. Comput. Chem.*, 2012, **33**, 580–592.
